# Dynamic Formation of Asexual Diploid and Polyploid Lineages: Multilocus Analysis of *Cobitis* Reveals the Mechanisms Maintaining the Diversity of Clones

**DOI:** 10.1371/journal.pone.0045384

**Published:** 2012-09-20

**Authors:** Karel Janko, Jan Kotusz, Koen De Gelas, Vera Šlechtová, Zuzana Opoldusová, Pavel Drozd, Lukáš Choleva, Marcin Popiołek, Marián Baláž

**Affiliations:** 1 Laboratory of Fish Genetics, Department of Vertebrate Evolutionary Biology and Genetics, Institute of Animal Physiology and Genetics, AS CR, v.v.i., Liběchov, Czech Republic; 2 Museum of Natural History, Faculty of Biological Sciences, University of Wrocław, Wrocław, Poland; 3 Research Institute for Nature and Forest (INBO), Brussels, Belgium; 4 Biogenomics, K.U. Leuven Research and Development, Leuven, Belgium; 5 Faculty of Natural Sciences, University of Ostrava, Ostrava, Czech Republic; 6 Department of Invertebrate Systematics and Ecology, Institute of Biology, Wrocław University of Environmental and Life Sciences, Wrocław, Poland; 7 Department of Biology and Ecology, Faculty of Natural Sciences, Life Science Research Centre, University of Ostrava, Ostrava, Czech Republic; Biodiversity Insitute of Ontario - University of Guelph, Canada

## Abstract

Given the hybrid genomic constitutions and increased ploidy of many asexual animals, the identification of processes governing the origin and maintenance of clonal diversity provides useful information about the evolutionary consequences of interspecific hybridization, asexuality and polyploidy. In order to understand the processes driving observed diversity of biotypes and clones in the *Cobitis taenia* hybrid complex, we performed fine-scale genetic analysis of Central European hybrid zone between two sexual species using microsatellite genotyping and mtDNA sequencing. We found that the hybrid zone is populated by an assemblage of clonally (gynogenetically) reproducing di-, tri- and tetraploid hybrid lineages and that successful clones, which are able of spatial expansion, recruit from two ploidy levels, i.e. diploid and triploid. We further compared the distribution of observed estimates of clonal ages to theoretical distributions simulated under various assumptions and showed that new clones are most likely continuously recruited from ancestral populations. This suggests that the clonal diversity is maintained by dynamic equilibrium between origination and extinction of clonal lineages. On the other hand, an interclonal selection is implied by nonrandom spatial distribution of individual clones with respect to the coexisting sexual species. Importantly, there was no evidence for sexually reproducing hybrids or clonally reproducing non-hybrid forms. Together with previous successful laboratory synthesis of clonal *Cobitis* hybrids, our data thus provide the most compelling evidence that 1) the origin of asexuality is causally linked to interspecific hybridization; 2) successful establishment of clones is not restricted to one specific ploidy level and 3) the initiation of clonality and polyploidy may be dynamic and continuous in asexual complexes.

## Introduction

In many animal and plant taxa sexual and parthenogenetic biotypes coexist (we refer to such systems as “asexual complexes”), which makes them ideal models to test the validity of theoretical predictions about the evolution of sex. For example, the predicted long-term disadvantage of asexuality seems supported by available phylogenetic data, which generally indicate that asexual lineages have short life spans [1]. Ancient clones, if existing, are often restricted to geographic regions where presumably fitter competitors, i.e. sexual and younger asexual counterparts, are absent (e.g. [2]). Because coexisting biotypes often comprise several ploidy levels, asexual complexes also provide an excellent opportunity to study the consequences of polyploidisation. The substantial fitness effects of polyploidisation seem corroborated by the fact that usually only one ploidy level is able to establish successful clones, or that one ploidy constitutes the dominant component with other ploidies occurring as ephemeral off-shoots [3], [4] or arising from exceptionally rare genomic combinations able to overcome the constraints of ploidy change [5].

Detailed investigations into some model asexual complexes have not only served as test-cases for theoretical predictions, but have also directly stimulated the formulation of other hypotheses about the evolution of asexuality and polyploidy. For example, the frequent association between parthenogenesis and hybrid nature of asexuals’ genomes led to the widely accepted interpretation that hybridization acts either as a stimulus for asexuality or is a prerequisite for its maintenance, or both [6], [7]. It has also been proposed that the initiation of parthenogenesis requires certain exceptional gene-to-gene combinations because many asexual taxa possess limited genetic variability compared to their sexual counterparts [3], [8] and many attempts of crossing proposed sexual ancestral species failed to produce clonally reproducing animals but instead produced either non-viable or sterile progeny or hybrids with ‘normal’ Mendelian segregation (rev in. [9]). Corley and Moore [10] observed difficulties in switching to asexual reproduction in a complex of parthenogenetic cockroaches and argued that such difficulties may explain the dominance of sex in eukaryotes in general (an analogous hypothesis has subsequently been raised by [11] and most recently by Stöck et al. [8] under the name “rare formation hypothesis”).

However, the generalizations of such hypotheses are still problematic as there are a large number of exceptions to almost any pattern. The polyphyletic origin of clones inferred in many complexes indicate that the induction of clonality may not be as exceptional as previously believed (see, e.g., [12], [13]). The hybrid state of contemporary parthenogens may not necessarily reflect the causal link between hybridization and asexuality, but may result from the accidental “freezing” of successful hybrid states through the disruption of meiosis subsequent to the hybridization (hybrid advantage hypothesis; [14]). In fact, there are cases of non-hybrid asexual animals even among vertebrates such as *Lepidophyma* [15]–[17].

Contemporary knowledge of the evolution of most asexual complexes clones is largely based on phylogenetic inference [18], which has limited power to identify causal mechanisms and disentangle neutral vs. non-neutral processes affecting the distribution and diversity of clones [1]. Although the clonal reproduction theoretically ensures that the reconstruction of gene-genealogies may be informative about the history of the whole evolutionary unit – the clone, the study of Kraus and Miyamoto [19] showed that genomic rearrangements or even rare recombination events can obscure the original phylogenetic signal. Sousa-Santos et al. [20] even suggested that assemblages of clones, which appear as polyphyletic with respect to their sexual ancestor, may represent descendants of a single clonal lineage which diversified by subsequent genomic introgressions. Angers and Schlosser [12] further demonstrated that even if the multiple formation of independent clonal lineages may be credibly inferred, the origin of asexuality might have been confined to a certain period in the past, with few or no events in recent times. To avoid these problems in addressing the processes maintaining the clonal diversity and the dynamics of asexual and polyploid biotype formation, phylogenetic investigations should be complemented by experimental studies of interactions among biotypes.

**Figure 1:**
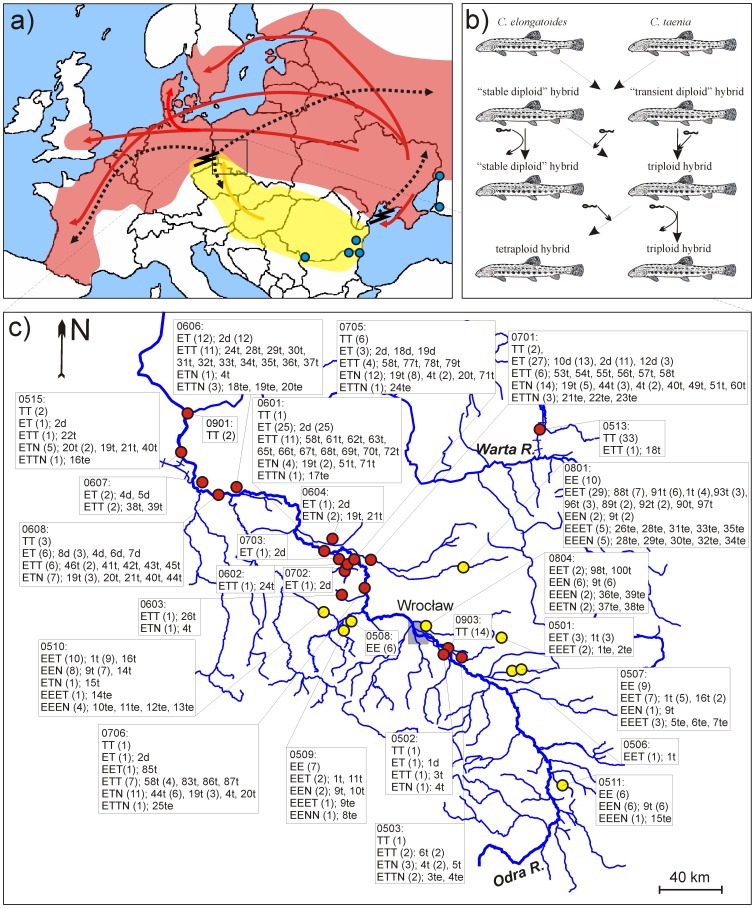
Europe-wide distribution, reproductive pathways and contact zone in Odra R. basin of studied species. (a) Distribution area of *C. tanaitica* (blue circles), *C. taenia* (red area), and *C. elongatoides* (yellow area), with the directions of postglacial colonization of Europe by *C. taenia* (red arrows) and *C. elongatoides* (yellow arrow). Secondary contact zones are indicated by zigzag symbols, and the dispersal of clonal lineages is indicated by dotted arrows (modified from [13], [46], [64]); (b) inferred reproductive pathways in *Cobitis* complex. Sperm at straight arrow indicates true fertilization, whereas sperm at round arrow indicates a triggering the egǵs development without paternal genetic contribution to the offspring; (c) Sampling sites in the Odra R. hybrid zone. For each locality, the presence of *C. taenia* and *C. elongatoides* is indicated by red and yellow dots, respectively and we indicate co-occurring hybrid biotypes, and their number in parentheses, if more than one. For each biotype on a given sample site, we list the presence of clones (MLL) and their absolute frequencies in parentheses, if more than one.

The *Cobitis taenia* hybrid complex of the bottom-dwelling spined loach could be used to address these issues. This complex comprises several sexual species of parapatric distribution, of which three species show closely adjacent ranges in non-Mediterranean Europe (although they have not been found syntopically): *C. elongatoides*, *C. tanaitica,* and *C. taenia* (Figure 1a). Mitochondrial DNA phylogeography suggested repeated reproductive contact of these 3 species during Pleistocene/Holocene climatic oscillations and indicated the formation of several hybrid lineages in the Central European and the Black sea shelf regions, which subsequently colonized most of the continent (Figure 1a; [13], [21]). Hybrids are mostly triploid, but di-, and tetraploid forms have also rarely been found in some regions. Laboratory experiments [22]–[24] proved the clonal gynogenetic reproduction of such hybrids. Hybrid females thus require a sperm from related sexual species to trigger the development of a clonal oocyte but the sperm’s genome is subsequently degraded. Nonetheless, the clonal egg may occasionally be fertilized, which results in polyploidisation (the Genome Addition Mechanism; Figure 1b; [24]). Wild-caught diploid hybrids produced mixed clutches of about 2/3 clonal diploid and 1/3 triploid progeny.

Phylogeny-based inference of multiple independent formations of clonal linages was recently supported by the successful synthesis of gynogenetic diploid and triploid hybrids through the laboratory crossing of *C. elongatoides* and *C. taenia* [9].This makes spined loaches an excellent model group among animals where initiation of asexuality can be studied in ‘real time’. However, it still remains unclear how often such crossing lead to successful establishment of naturally occurring clones, because although all “freshly” synthesized diploid F1 hybrids produced clonal eggs, most of them produced all-triploid progeny. Although their 3n progeny is fertile (unpublished data of the authors), such diploid biotypes may not establish persistent diploid clones and we refer to them as “transient diploid” hybrids [9]. Only one in nine F1 females resembled the wild diploid hybrids in that she produced a mixed clutch of di- and triploid progeny and had the potential to establish diploid clonal lineage.

Moreover, it is likely that polyploidisation may affect the relative performances of diploid and polyploid hybrid biotypes since significant differences in the standard metabolic rates between diploid and triploid spined loaches have been reported [25]. Hence, given the Europe-wide dominance of triploid hybrids it is unclear how dynamic the recruitment of clonal lineages is in nature and whether the formation of persistent clonal lineages is restricted to triploids only.

In this paper, we build upon the results of previous phylogeographic and experimental studies and perform population genetic analysis of Central European hybrid zone between *C. taenia* and *C. elongatoides* in order to answer five questions. First, we consider whether the induction of asexuality is strictly bound to interspecific hybridization, or whether spontaneous asexuals may also be detected. The appearance of the latter might support the hypothesis that parthenogenesis arises frequently, but might only be favored by selection when coupled with the hybrid state [14]. Second, we consider whether clonally reproducing hybrids are the only form of hybrids in this complex, or whether hybrids with Mendelian segregation exist too. The latter may indicate that the formation of clones requires some specific allelic combinations, whereas most hybridization events would lead to unstable hybrid biotypes (e.g. [8]). Third, we question whether the ability to form clonal lineages is confined to a particular level of ploidy, or whether successful clones belonging to several ploidy levels originate continuously. Fourth, we evaluate whether the induction of asexuality was an exceptional event, or whether clonal lineages arose on multiple occasions, even at the micro-scale of a single river catchment. Finally, fifth, we investigate whether new clones originate *de novo* continuously, or whether their recruitment was confined to a certain period in the past (as in the *Phoxinus eos-neogaeus* complex [12]).

## Methods

### Ethics Statement

Fishing and tissue collection was performed under the permissions of the Polish Government No. DLOPiK-op/ogiz-4200/V-13/4443/06/aj, DLOPiK-op/ogiz-4200/V-11/7656, 9940/07/08/łw and No. DOPozgiz-4200/V-27/1612/09ls.

### Sampling and Genotyping

We visited all sites in middle Odra basin where Cobitis fishes have been reported in the literature, in addition to many new locations between 2005 to 2009 (Figure 1c, Table S1) and collected samples (finclips) from 27 sites (420 individuals) distributed along the Odra river basin from the 683rd to 146th km above the mouth of Odra R. In the lower part of the basin, only *C. taenia* has been reported so far [26], and a hybrid zone was not expected to be present.

#### Identification of genomes

Janko et al. [21] reviewed genetic markers, whose combination allows the identification of species-specific genomes found in central Europe; specifically, *C. taenia* (diploid genotype: TT), *C. elongatoides* (EE), *C. tanaitica* (NN), and their hybrids. In order to identify the genomes of sampled specimens (including pure species and hybrids) we used diagnostic allozyme loci Gpi-A (glucose-6-phosphate isomerase, EC 5.3.1.9), sAat (aspartate aminotransferase, EC 2.6.1.1), sMdh-A (malate dehydrogenase, EC 1.1.1.37), and Pgm (phosphoglucomutase, EC 2.7.5.1), as in [21]. We also sequenced first intron of the nuclear S7 gene to confirm genomic constitution in doubtful cases according to [21]. Thus, we could define diploid (ET), triploid, and tetraploid hybrids that comprised different combinations of T, E, and N genomes.

#### Microsatellite analysis

In total, 10 microsatellite loci were analyzed for genetic polymorphism under the routine conditions [9], [27]. Samples from localities 0501 to 0707 were analyzed on all 10 loci, while samples from localities 0801–0903 were analyzed in a different laboratory, and were not scored for locus cota037, since there were problems with the analysis of this locus. Nonetheless, the scorings of all other loci were cross-validated among both groups of samples. To obtain the highest possible power of resolution, all subsequent analyses were performed on samples from localities 0501 to 0707, using all 10 loci. Only the delimitation of clonal lineages (see below) was performed for both datasets to obtain maximal geographical coverage to evaluate the distribution of clonal lineages.

#### Ploidy estimation

Where available, ploidy was estimated by using the erythrocyte measurement [28]. In allozymes, the ploidy estimation was based on the gene dose effect, which was validated by flow cytometry as suitable for differentiating between the diploid and triploid forms of spined loaches [24]. Finally, we determined the level of ploidy in microsatellites by counting the maximum number of alleles per locus.

#### Mitochondrial DNA sequencing

We amplified a 1190 bp fragment of the cytochrome b (cyt b) gene according to [29] in all sexual individuals, and 1 representative of each multilocus genotype (MLG) for the hybrids. We did not sequence tetraploids for financial reasons. Phylogenetic relationships among mtDNA haplotypes, including those found in previous studies [13], [30] (accession Nos: AY706159–AY706203; EU262736) as well as the new ones (accession Nos: JX402883- JX402904) were estimated using the statistical parsimony [31] which was implemented in the TCS Program, version 1.06 [32].

### Evaluation of Mendelian Hybridisation and Asexual Reproduction

To detect possible post-F1 diploid hybrids, which would indicate the possibility of non-clonal reproduction of hybrids and of introgressive hybridization, we applied the NewHybrids software [33]. Since clonal reproduction may violate the assumption of no linkage among markers, we performed this analysis in three ways: we first analyzed all individuals; we subsequently used only one representative of each unique MLG and finally, when several MLGs formed one multilocus lineage (see below), we used only one representative of each such lineage. The latter two analyses removed the effect of clonal propagation of identical genomes. We also repeated those analyses with either Cota_06 or Cota_041 locus removed, since DeGelas et al. [27] reported possible linkage between those loci.

The inbreeding coefficients F_IS_ were also estimated for sampled diploid biotypes (both parental species and their putatively clonal hybrids) since significantly negative values may indicate clonal reproduction [34]. Finally, the existence of clonal reproduction was inferred from observations of individuals sharing the identical genotypes (see the next paragraph).

### Delimitation of Clonal Lineages

Individuals with the same MLG may represent members of the same clone (i.e. the family descending from single event of switch from sexual to asexual reproduction). However, depending on the variability of markers, identical individuals may also arise by chance from independent sexual events. To disentangle these alternatives, we used GeneAlex 6 [35] to calculate the probability that two randomly selected individuals have the same genotype at multiple loci (probability of identity; PID). This calculation was completed for varying combinations of loci, ranging from a single locus to all ten loci. We further employed the GenClone software [36] to calculate the probability that observed multiple copies of the same MLG arose by independent sexual events (P_sex_). P_sex_ is estimated from the probability of each genotype occurring in the pool of all diploid individuals (Pgen_(FIS)_; [37]) taking into account deviations from the Hardy-Weinberg equilibrium. GenClone was also used to perform a Monte Carlo permutation to evaluate the maximum number of unique MLGs that may be resolved by all possible combinations of one to ten loci. Due to software limitations, these calculations were used for diploid individuals only. However, given higher number of allelic combinations in triploids and tetraploids, their resolution power should be even greater in higher ploidy levels.

**Figure 2:**
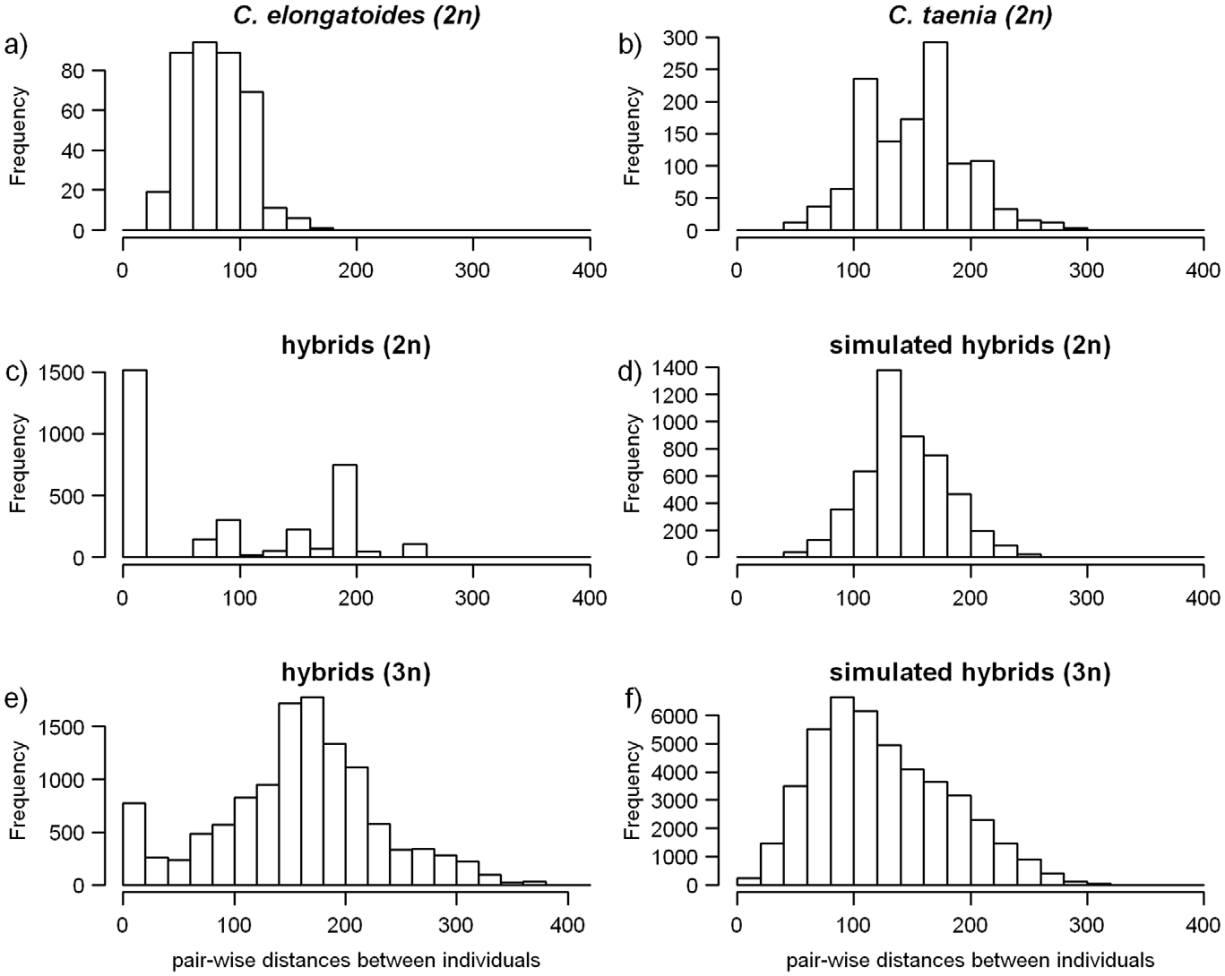
Histograms of the frequency distribution of pairwise distances in bp among genotyped individuals. (a–b) Data from *C. elongatoides* and *C. taenia*; (c–d) Distributions from observed (natural) and simulated diploid hybrids; (e–f) distributions from the observed (natural) and simulated triploid hybrids.

While some MLG may represent distinct clones, others may differ by scoring errors and/or somatic or post formational mutations and form the so called ‘multilocus lineage’ (MLL). Such MLL should represent evolutionarily independent clones. In order to distinguish which MLG form the same MLL, Rogstad et al. [38] suggested to construct the pairwise distance matrix and inspect the histogram of pairwise comparisons among all individuals (Figure 2; [38]). While the shape of such histogram may depend on various processes, the presence of MLG pairs with extremely low differences, probably representing the same MLL, should produce an initial peak close to zero. Douhovnikoff and Dodd [39] further proposed an objective method to place the threshold distance under which any two MLG should be grouped into one MLL. The threshold is based on relatedness within and among known clonal lineages. Since such knowledge is usually not available [40], we proceeded in a following way: We first calculated two distance metrics to reconstruct the distance matrix (we used the number of distinct alleles or the sum of the differences in allele lengths between each pair of MLG [40]). Then, we simulated hybrid genotypes by random combination of individuals from hybridizing species to obtain the null distance distribution. Using the sequential Bonferroni’s correction for multiple comparisons, such a null distribution allowed us to evaluate the probability that any two MLG belong to the same clone (for details see the online supporting material; Text S1, Figure S1). The calculations were performed on ten microsatellite loci using samples from localities 0501 to 0707, as well as on a dataset of nine loci using all samples. From now on, we shall use the terms ‘clone’ and ‘MLL’ as equivalent unless stated otherwise.

### Relative Ages of Clones

Obtaining appropriate estimates of divergence times of clones based on nuclear variability is not trivial, since available software usually do not allow mixing of different ploidy and do not assume asexual reproduction. We applied several approaches based on measures of allelic differences between all MLL and their ancestors to obtain the relative estimates of ages of distinct MLL. For diploid hybrids, we assumed both hybridizing species as their ancestral biotypes. For triploids, we assumed both species as well as sampled 2n hybrids as potential ancestral biotypes. To account for geographical variation in the diversity of sexual species, we also included the microsatellite data from specimens used in De Gelas et al. (2008) that originate from Belgium (*C. taenia*) and the Danube River (*C. elongatoides*).

We used the PopDist software [41] to calculate Tomiuk and Loeschcke’s [42] identity measure (*I*) between each MLL and its ancestors. This measure is particularly suited for analyses of populations differing in reproductive mode and ploidy since it examines the sharing of alleles rather than gene frequencies and has previously been applied to other asexual complexes including hybrid and polyploid biotypes [5]. The value of *I* is proportional to the evolutionary time since the divergence of asexual population from its ancestors and therefore it may be used to measure relative ages of observed clonal lineages. However, since PopDist assumes a single ancestral population, whereas *Cobitis* asexuals apparently originated through mixing of genes from two ancestral species, we coded each diploid MLL as a distinct population, and calculated its identity with a pooled population of all sexual individuals (*C. elongatoides* and *C. taenia*). Since triploids originate from the incorporation of sperm into the ovum of the clonal diploid, we calculated the age of triploids by pooling both sexual species and diploid hybrids into a single population, and then calculated *I* for each triploid MLL. We did not calculate *I* for tetraploids, since their ancestors are both diploids and triploids, which cannot be pooled into a single population for PopDist.

Due to possible problems with the calculation of *I* in hybrid complexes, we further calculated another two estimates of the age of clones, hereafter referred to as *dist.mut* and *dist.bp*, which rely on different assumptions. Their applicability has been evaluated by computer simulation (see below). Assuming that post-formational mutations produce new alleles, *dist.mut* estimates the distance between each MLL and its ancestors by simply using the average number of alleles sampled in a given MLL that were not present in either of its ancestral species. On the other hand, *dist.bp* evaluates the sum of base-pair (bp) length differences between each MLL and the set of closest alleles sampled in its ancestors. *dist.bp* therefore assumes that more similar alleles are more closely related to each other. Note, that in the case of triploids, we calculated distances from both sexual species and the 2n hybrids that represent the maternal ancestor of triploids (similarly, in the case of tetraploids, we assumed that their ancestors were either sexual species or triploid hybrids). When the number of allelic copies was indistinguishable in some triploids, we proceeded in two ways: we either assumed that such triploids possess the 2 alleles that produce the least difference from their ancestors or we assumed that they possess the 2 alleles, which maximize the difference. The former approach is conservative, since it results in lower age estimates in such problematic cases. We multiplied the values estimated in triploids by 2/3 to make the estimate comparable to diploids, taking into account higher number of gene copies in the genomes of polyploids [43] (the same calculations for tetraploids were multiplied by 1/2).

An Individual-Based simulation Model (IBM) was used to evaluate the suitability of both indices to estimate the age of clones; see Text S1 and Figure S1 for details. In brief, we modeled the evolution of microsatellite variability of two sympatric parental species. The simulations were run under varying per-generation mutation rates from 10^−6^ to 10^−2^ covering the mutation rates in related family Cyprinidae [44]. To account for the effects of geographical structure, both species were structured into two regions (demes) interconnected by a per-individual migration rate *m*, which varied so that the average effective number of migrants (*M*) ranged between 1 and 0.01 per generation. Simulated diploid and triploid clones arose from hybridization of randomly chosen pairs either within the region 1 or the region 2. The simulations were performed under varying mutation rates and models (including stepwise as well as K-mutation model) as well as under varying migration rates among demes of parental species. Each simulation ran for 40000 generations and after every 200 generations, we calculated *dist.mut* and *dist.bp*. Each index was calculated in two ways. First, by comparing the alleles of a given clone against those in its ancestral sexual deme. Second by comparing it with the alleles in the allopatric sexual deme. The latter way tested the reliability of *dist.* estimators in the case of incomplete sampling of sexual ancestors of clones. To evaluate the suitability of those indices for age estimation, the relationships between each index and age were tested by a linear mixed effect model where the clones were considered as factors with random effects and age as a fixed factor. The explanatory variable was modeled as a second-order polynomial.

#### Temporal dynamics of clonal recruitment

We tested the null hypothesis that all clones are of the same age (e.g. as if their origins are restricted to a brief period when expanding parental species met, [12]). Under the null hypothesis, we may assume that the observed variability in age estimates among MLL results from the stochasticity of coalescent and mutation processes. Therefore, the distribution of observed indices should vary around the true age common to all clones. Rejection of the null hypothesis would mean that observed clones truly differ in their ages and have been arising rather continuously during longer period. To test this, we first reconstructed histograms of *dist.mut* and *dist.bp* of simulated clones from every 200th generation of the simulation runs described in the previous section. For each histogram we calculated the corresponding *D* values of Hartigan’s dip test of unimodality [45] to obtain the distribution of *D* under the null hypothesis that all clones are of the same age. In real situation, the *dist.mut* and *dist.bp* is likely to be estimated from limited geographical sample of sexual ancestors, whereas some of the analysed clones might have originated in unsampled ancestral regions. Such allochthonous clones would falsely appear as old due to the presence of unsampled ancestral alleles. In order to account for incomplete geographical sampling, we also combined simulated clones from both regions in varying proportion and calculated the *dist.mut* and *dist.bp* values against the sexuals from the first region only.

Finally, we compared Hartigan’s *D* from the observed data to the simulated values. The significance of deviations from unimodality was evaluated as the proportion of cases when the simulated values produced a *D* value equal to or greater than the observed one. High values indicate that the age estimates obtained from the observed array of clones are significantly more heterogeneous than expected under the null hypothesis. The *D* values were calculated by using the R software (R development Core Team 2011).

## Results

### Sampling and Genotyping

Scoring of allozyme loci and S7 sequence variability in cases where the distinction of genomes was problematic led to the identification of the following biotypes (Table S2, Figure 1c): (1) diploids with 38 individuals *C. elongatoides*, 66 of *C. taenia*, and 81 of ET hybrids; (2) triploid hybrids with some combinations of *C. elongatoides*, *C. taenia*, or *C. tanaitica* genome (55 EET, 54 ETT, 25 EEN, 62 ETN); and (3) tetraploid biotypes (12 EEET, 12 EEEN, 14 ETTN, and 1 EENN). Note, that in several cases we were unable to determine the precise proportion of respective genomes in tetraploid individuals (Table S3).

Within the Odra region we observed no significant deviations from the Hardy-Weinberg equilibrium in any locus when analyzing the single population of *C. taenia* with a sufficient number of samples (locality 0513). When we pooled all parental individuals into the species-specific datasets, we observed no deviations from HW in *C. elongatoides,* but in *C. taenia* the locus cota_006 showed a significant lack of heterozygotes, resulting in significantly positive F_IS_.

**Figure 3:**
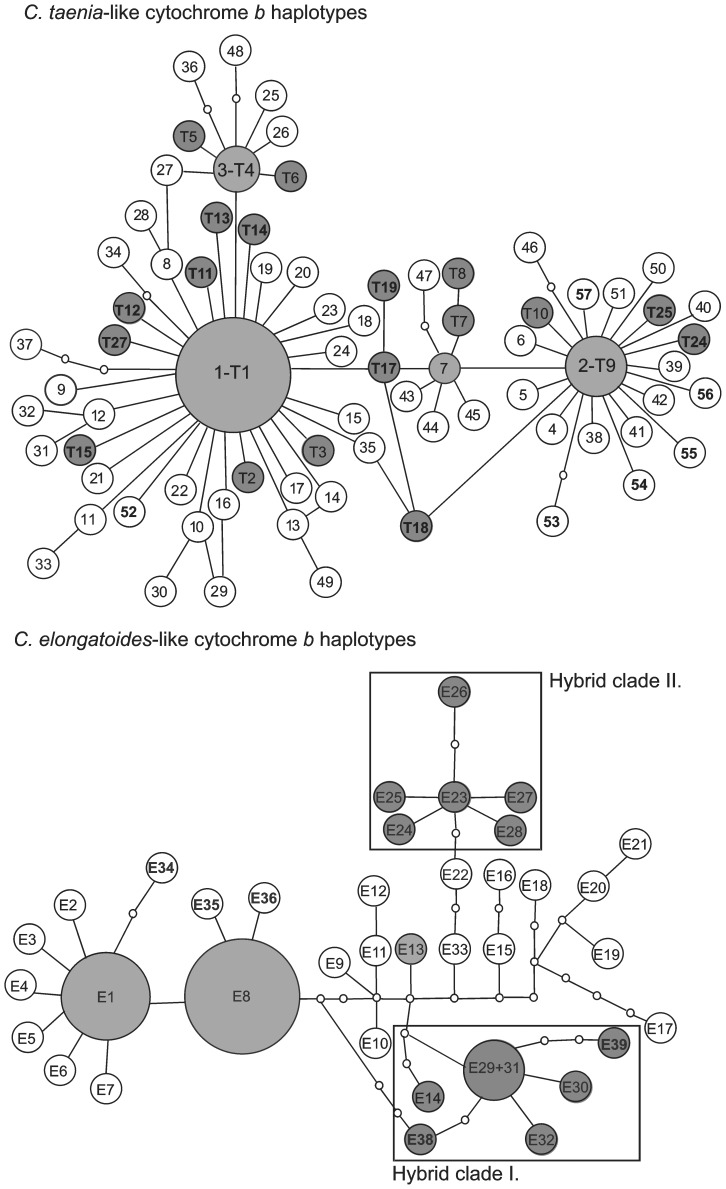
Unrooted statistical parsimony networks of haplotypes belonging to *C. taenia*-like (T) (upper panel) and *C. elongatoides*-like (E) (lower panel) clades (sensu [**13**]). White circles denote haplotypes found in sexual individuals only, dark grey circles denote those found in hybrids only, and light grey circles denote haplotypes shared by both hybrid and sexual individuals. The sizes of haplotypes are proportional to their frequency. Small blank circles represent missing (unobserved) haplotypes. Newly sequenced haplotypes are in bold. Rectangles delimit the hybrid clades I and II.

TCS grouped whole mtDNA variability into 2 networks, 1 corresponding to *C. elongatoides*, and the other corresponding to *C. taenia* clusters. In comparison to previous studies, we found 17 new haplotypes that were *C. taenia*-like and five new haplotypes that were *C. elongatoides*-like. All EEN triploids, as well as few ET diploid hybrids, possessed *C. elongatoides*-like haplotypes, while all remaining hybrids possessed *C. taenia*-like haplotypes (Table S2). Most hybrids shared their haplotypes with the parental species; however, some hybrids possessed haplotypes that were different from their sexual ancestors. Even in such cases, the distance between “sexual” and “hybrid” haplotypes was low (usually no more than a single mutational step apart; Figure 3). The EEN hybrids were exceptional, since their haplotypes belonged to more substantially diverged mtDNA lineage reported by [13] from other parts of Europe too.

### Evaluation of Mendelian Hybridisation and Asexual Reproduction

Analysis using the NewHybrids showed no evidence of post-F1 hybrids. In all types of analyses all diploids might be unambiguously determined as either *C. elongatoides, C. taenia*, or their F1 hybrids with a posterior probability higher than 0.99. We did not observe any MLG shared by two or more individuals in *C. elongatoides* or *C. taenia*. In contrast, all loci showed significantly negative F_IS_ values in diploid hybrids. Twenty MLG were shared by two or more individuals among 2n or 3n hybrids (additional five multicopy MLGs were found at locality 0801 using nine loci only). In all cases of multicopy diploid genotypes, the P_sex_ was negligible (<10^−2^), suggesting that distinct individuals with the same MLG belong to the same clone. While all tetraploids also appeared as fixed heterozygotes for species-specific diagnostic markers, no tetraploid MLG were shared by two or more individuals.

### Delimitation of Clonal Lineages

The summary of microsatellite variability is provided in Tables S4 and S5 The probability of identity of diploid genotypes was negligible (in the order of 10^−6^), and the Monte Carlo procedure showed that a set of five loci is sufficient for the accurate determination of all distinct MLG among sampled diploid individuals.

The frequency distribution of the pairwise distances was similar for both of the distance metrics used in this study. The results for *dist.bp* are shown in Figure 2. We observed more or less symmetric distributions with no peak at or close to zero for *C. elongatoides* and *C. taenia*, as well *as* in the simulated datasets of diploid and triploid hybrids. The datasets of natural diploid and triploid hybrids differed notably from these diagrams, since we observed bimodal distributions with the first prominent peak located at zero. Comparison of the simulated and observed frequency distributions (see above) detected 15 groups of diploid or triploid MLG that had significantly small distances after the sequential Bonferroni’s correction (Table S2). These MLG were then grouped into respective MLLs and considered as clone-mates. This was confirmed by the negligible P_sex_ values calculated on the set of loci that were identical among grouped diploid MLG. Seven of these MLL comprised hybrids with E and T genomes, six comprised trigenomic hybrids with E,T, and N genomes, and one comprised hybrids of E and N genomes. Individual MLL, as well as the remaining MLG, were subsequently considered as independent clonal lineages (also referred to as “clones”).

The power to resolve hybrid MLGs slightly decreased when using 9 loci, but the delimitation of MLL did not change when using samples from localities 0501–0707, or when using the full dataset scored on nine loci only (data not shown). Most hybrids from localities 0801 and 0804 belonged to independent clonal lineages, but some individuals were attributed to MLL 1 and 9 (see Table S3).

We noted that allelic variation of 30 triploid MLG could be decomposed into a diploid set, which is identical to some of the sampled diploid hybrids and an additional haploid set of alleles (Table S2). Therefore, we hypothesize that such pairs of 2n and 3n MLG represent diploid ancestors and their polyploid derivatives. Similarly, 24 out of 39 tetraploid MLG possessed triploid sets of alleles, which have been identified in some of the sampled triploids.

### Relative Ages of Clones

**Figure 4:**
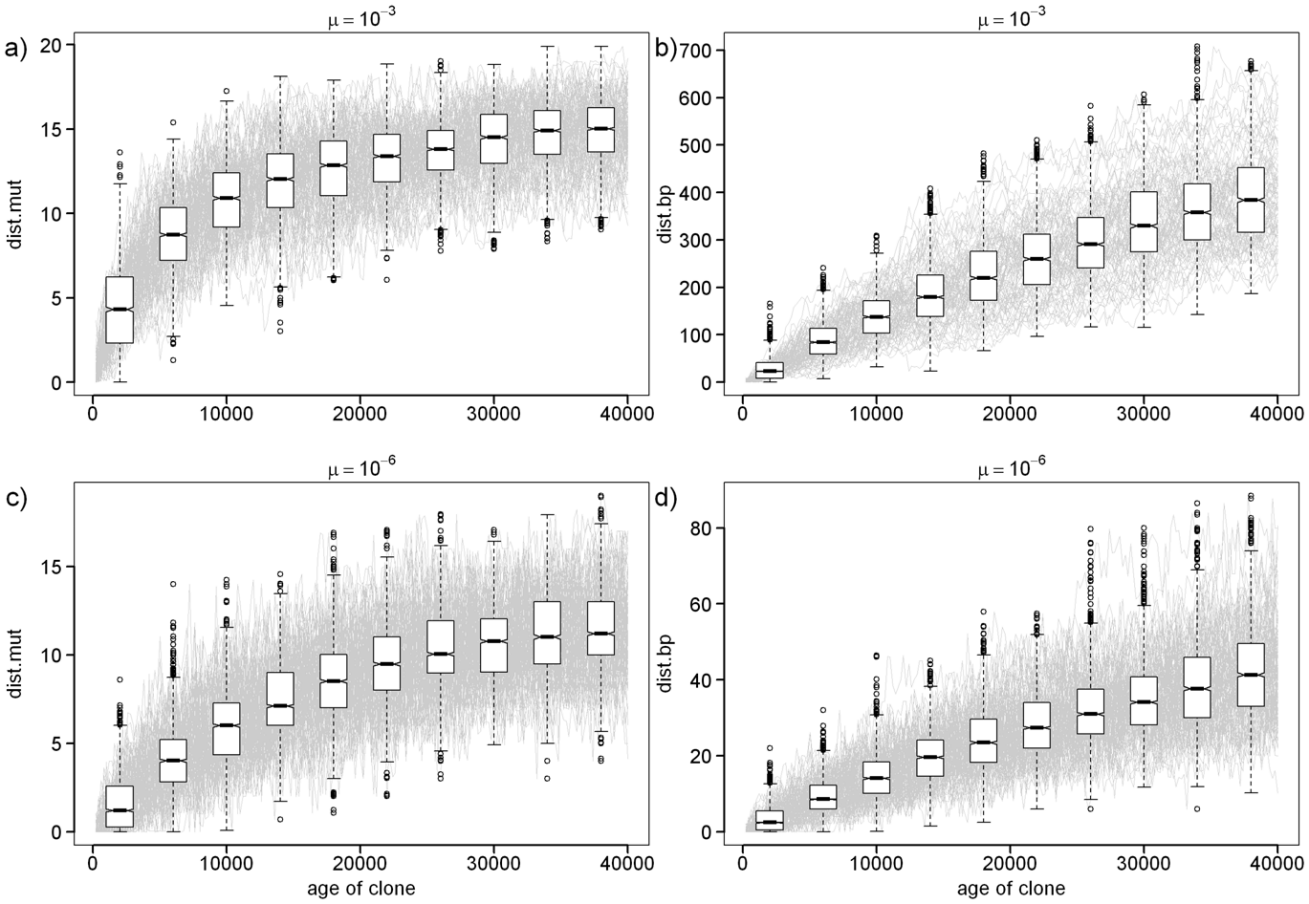
The plots of *dist.mut* and *dist.bp* values as a function of true age. Each gray line tracks the evolution of one clonal lineage over the time. Simulations are shown for the 2 values of mutation rate (µ) under SSM mutation model. At selected times, we represent the boxplots showing the median of simulated values of *dist.mut* and *dist.bp*. as well as first and third quartile; whiskers represent 1.5 times the interquartile range.

Simulation demonstrated that both *dist.mut* and *dist.bp* correlate highly significantly positively with the true age of the clone under varying combinations of mutation rates and models (Linear mixed model, p<<0.001; Figure 4). The use of second-order multinomial is justified since the correlation is not strictly linear as the signal becomes saturated with the increasing age of the clone and both indices tend to plateau. Obviously, the time required to reach such a plateau depends on the mutation rate and also, in the case of *dist.mut*, on the number of loci analyzed (and hence the maximum possible numbers of allelic differences). The time required to reach the plateau is much longer for *dist.bp* measures since it depends not only on the presence/absence of shared alleles but also takes into account the genealogical distance between alleles.

**Figure 5:**
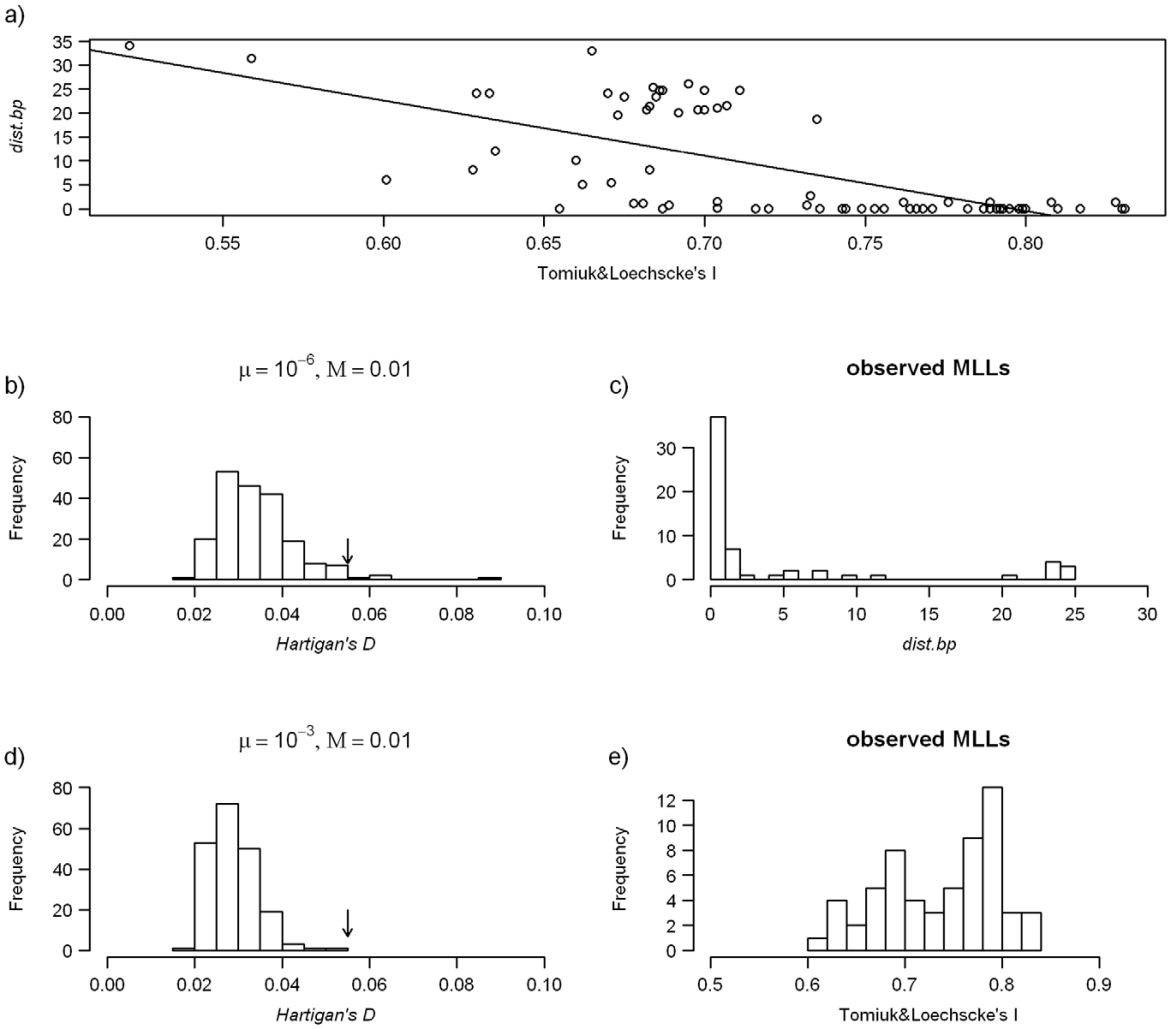
Observed and simulated distribution of clonal age estimators. (a) Correlation between the observed values of *dist.bp* and Tomiuk and Loeschcke’s *I*; (b, d) Distributions of Hartigan’s *D* values calculated from simulated *dist.bp* histograms at every 200^th^ generation of the simulation. Each value was calculated from a 50∶50 mixture of sympatric and allopatric clones. Arrows indicate the observed value; frequency distributions of the observed values of *dist.bp,* and *I*, respectively (c, e).

Although applied age estimators (i.e., *dist.mut, dist.bp* and *I*) rely on different metrics and assumptions, they were significantly correlated with each other (R^2^ ranging from 0.45 to 0.92; p<10^−5^; Figure 5). This was true for all asexuals, as well as for the separate analysis of *C. elongatoides* – *C. taenia* clones or triploid clones only. Clones with the *C. tanaitica* genome were characterized by relatively high values of *dist.mut* or *dist.bp* estimates and low *I* values, which most likely reflects the fact that *C. tanaitica* samples were not included in our study. In comparison, the majority of clones combining *C. elongatoides* and *C. taenia* genomes possessed the full set of alleles that were identical to those occurring in their ancestors. This resulted in zero values for *dist* estimates and in high values of *I*. Nevertheless, a substantial proportion of the clones possessed unique alleles, making their *dist* estimates non-zero, and driving *I* to lower values.

The distribution of age estimates for clones with *C. elongatoides* and *C. taenia* genomes showed a clear multimodal pattern. The observed Hartigan’s *D* values (0.082 for *dist.mut* and 0.054 for *dist.bp*; Figure 5) were compared against simulations under varying mutation and migration rates and were always higher than at least 98% of simulated values (depending on the simulated mutation and migration rates, Figure 5). It is notable that observed *D* values were significantly high even when the null distributions of indices were calculated against a single sexual region from a 50∶50 mixture of sympatric and allopatric clones. The same holds true for the *D* values that were calculated separately for diploids and triploids (not shown). Similarly, the Hartigan’s *D* values were significantly high when assuming the maximal possible distance of triploids with unresolved dose of alleles (results not shown). This indicates that the distribution of observed age estimates deviates from the one expected under the hypothesis that all clones have the same age, even when taking into account the geographical structure of parental species and potential effect of incomplete sampling. Similarly, Tomiuk and Loeschcke’s identity estimates for *C. elongatoides* – *C. taenia* clones apparently deviated from unimodality (Figure 5). Overall, this result supports the hypothesis that sampled clones differ in true age.

## Discussion

### Hybridization and Origin of Asexuality

*C. elongatoides* and *C. taenia* are widespread throughout the Odra R. basin, but were never found to co-occur, conforming to previously reported parapatric distribution of both species on a continent-wide scale [21]. *C. taenia* mostly occupied the main river but also entered into some inflows, while *C. elongatoides* occurs exclusively in smaller tributaries. Such a pattern may reflect competitive exclusion resulting from different habitat preferences but more detailed study is necessary to test it. In any case, reproductive contact between the species is likely, since the distance between the localities occupied by both species is small, and absent of any obvious obstacles for dispersal (Figure 1c). Haplotypes from *C. elongatoides* and *C. taenia* belong to phylogroups that expanded from Panonian and Pontic refugia, respectively [13], [46], which is consistent with the hypothesis that both species got into secondary reproductive contact in the Central Europe after the last glacial maximum (LGM).

Cytochrome b analysis implies polyphyletic relationships of hybrids to the parental species, indicating that diploid and polyploid hybrids arose upon multiple occasions. The presence of both *C. elongatoides* and *C. taenia* haplotypes in ET hybrids indicates reciprocal hybridization between both parental species. The delimitation of MLL as independent evolutionary units (clonal lineages) is validated by cyt *b* variability for hybrids containing E and T genomes; all members of the same MLL mostly shared the same haplotype or at least formed monophyletic mtDNA clusters with respect to the sexual ancestor (Table S2, Figure 3). However, the results were less satisfactory for hybrids that possessed the nuclear genomic component derived from *C. tanaitica*. Although MLL 9t appeared as monophyletic, some of the MLL combining E, T, and N genomes appeared as polyphyletic on the cyt *b* marker (e.g., MLLs 44t and 71t). This pattern probably results from the fact that *C. tanaitica* samples were not available for the simulations of pairwise distance distributions and the equivalence of ‘clone’ and ‘MLL’ should be considered with caution in such cases.

Haplotypes from all EEN triploids (MLLs 9t, 10t, 14t) form a monophyletic cluster corresponding to the ‘hybrid clade I’ of Janko et al. [13], which is widespread over Europe and contain hybrids carrying one genome of *C. elongatoides*, one of *C. tanaitica* and an additional third genome from either *C. elongatoides, C. taenia* or *C. strumicae* depending on the region [30]. Based on the combination of mtDNA and allozyme markers it has been proposed that the origin of the ‘hybrid clade I’ largely predates Holocene [13], [30] and involves initial *elongatoides* - *tanaitica* hybridisation followed by several independent triploidisation events. This asexual lineage probably survived the LGM in the Panonian basin, and co-expanded into Central Europe with *C. elongatoides*. Due to unknown microsatellite variability of *C. tanaitica* we can not exclude the possibility that MLL 9t, 10t and 14t belong to single evolutionary clone, which diversified too substantially to be pooled into single MLL by our method.

Divergences from sexual species were lower in all remaining hybrid biotypes, suggesting a recent and probably post-glacial, origin (Figure 3). The origin of ETN triploids may involve hypothetical relict *C. tanaitica* populations from the Odra region. However, *C. tanaitica* was not encountered in this study and most ETN hybrids possessed haplotypes related to, but absent from local, *C. taenia* populations. Therefore, we consider the allochthonous origin of ETN clones as more plausible. The absence of *C. tanaitica* mtDNA in these lineages may indicate unidirectionality of hybridization (see [29] for discussion of this issue) or a secondary addition of N genome to original ET hybrids. On the other hand, all clones without the *C. tanaitica* genome either shared cyt *b* haplotypes with local sexual populations, or diverged by single mutations (Table S2, Figure 3). Only MLL 86t and 55t possessed haplotypes that were sampled by Culling et al. [46] from *C. taenia* inhabiting the Dniester and Western Bug Rivers but were not found in the Odra R. However, even in this case, both haplotypes belong to the *C. taenia* clade, which is present in central Poland. It appears therefore, that the Odra hybrid zone is of postglacial origin and the sampled asexual populations are composed of clones of local (most of the clones containing E and T genomes) and allochthonous (EEN and possibly ETN clones) origins.

Data on *Cobitis* offer three-fold evidence for tight (and most likely causal) link between hybridization and asexuality. First, crossing between randomly selected individuals of both parental taxa directly lead to clonally reproducing females [9]. Second, asexuality of natural hybrids is evident not only from reproductive experiments [24] but also from the existence of several abundant hybrid MLG or MLL, as well as from significant heterozygote excess (negative F_IS_) among diploid hybrids, which are typical patterns for clonal populations [34]. Third, it seems that spontaneous asexuality is either absent or very rare since identical individuals or groups of significantly closely related MLG were never found among *C. elongatoides* or *C. taenia* samples. Moreover, both species either do not deviate from HW equilibrium or show heterozygote deficiency (pooled samples of *C. taenia*), which is typical for the Wahlund effect in structured populations, but not for clonal populations. Thus, the *C. taenia* complex provides compelling evidence in favor of the hypothesis that hybridization is causally related to the initiation of asexuality, which contrasts with the hybrid advantage hypothesis (reviewed in Kearney et al. [14]).

The absence of backcross or post-F1 hybrids together with previous experimental evidence for gynogenesis in studied hybrids strongly implies that hybrids with ‘normal’ Mendelian segregation are either absent or very rare. This sharply contrasts with other complexes, where the induction of clonal reproduction is restricted to only specific crosses while most other crossings result in ‘normal’ sexual hybrids [47], [48]. The initiation of asexuality in loaches appears as widespread phenomenon, which is not restricted to some particular crosses.

### Evolution of Polyploidy

Experimental data proved the production of clonal gametes in di- tri and tetraploid hybrid spined loaches [9], [23], [24], which is a necessary but insufficient prerequisite for establishment of persistent clonal lineages. If we define as evolutionary successful those clones, which are able of self-reproduction and of spatial spreading, then the existence of several diploid and triploid clonal lineages that were sampled in more than one specimen or were geographically widespread (Table S2) implies that successful clonal lineages may be recruited from at least two ploidy levels. In comparison, the ability to establish persistent clones in tetraploids is less evident since all tetraploid MLG occurred as single copies. However, even tetraploids might occasionally establish widespread clones, as evidenced from the high abundance of single 4n clone in the Volga Basin, Russia [49]. Available data on loaches therefore provide compelling evidence for the ability of several ploidy levels to establish successful clonal lineages upon many occasions.

The genomic composition of 34 triploid MLG and 24 tetraploid MLG could be derived from seven diploid and eleven triploid clones, respectively. This implies that such “derivable” clones descended from sampled clonal ancestors and is supported by sharing of mtDNA haplotypes between “derivable” 3n clones and their putative 2n ancestors. The exception was the triploid clone 41, whose haplotype differed from its diploid ancestor 6d by single mutation; yet, both haplotypes were in a monophyletic relationship. This is consistent with the hypothesis that polyploidy in spined loaches is achieved by sperm-incorporations into unreduced ova [24].

If the ability to form polyploids were restricted only to certain “predisposed” lineages, such lineages would be at disadvantage relative to other clones since part of their reproductive potential would be invested into production of higher ploidy levels rather than of their own clonal replicates. However, we observed that total abundance of a given diploid or triploid clone in our sample significantly correlates with the number of its polyploid derivatives (GLM with Poisson distribution of error; p<0.01). It appears therefore that polyploidisation is common to all or most clonal lineages. Together with results of laboratory breeding experiments, we conclude that most of ‘fresh’ F1 hybrids vanish a after single generation due to the production of all-triploid progeny, but those which can produce diploid progeny probably do not differ largely in the fertilization rate and may become established in longer term (Figure 1b).

On the other hand, it seems unlikely that some of 2n or 3n clones originate through ploidy reductions from parents with higher ploidies since genome exclusions have never been observed in crossing or cytological studies of European loaches. Moreover, the absence of diploid hybrids in regions outside the hybrid zones [21] suggests that triploids apparently didn’t reduce their genome there. Altogether, the high incidence of “derivable” polyploids, which constitute about 40% of 3n and 60% of tetraploid clones, suggest that frequent polyploidisation events constitute significant input to the genetic diversity of polyploids.

Polyploidization affects many traits, such as mutational load or resistance to parasites [50]. A general observation from most asexual complexes that persistent and successful clones are constituted by single ploidy level [3] suggests that polyploidization may have a substantial effect on evolution of clones. Reported complexes, where asexual lineages of various ploidy coexist (rev. in e.g. [43]) do not necessarily violate this assumption, since such cases often harbour diploid and polyploid forms that reproductively depend on one another, differ in genetic background (combine genomes from different sexual species), or one ploidy level constitutes the dominant form, with others occurring as ephemeral off-shoot ([4], rev. in [24]). In some well-studied cases it has been shown that coexistence of clones with different ploidy is possible only due to ongoing recruitment of the less fit type [4], or due to exceptionally rare genomic combinations overcoming the effects of ploidy change [5]. The lesson from spined loaches offers a different picture; despite the obvious effect of polyploidisation on physiology [25] and fecundity [51], the ability to establish successful clones is not ploidy-restricted and clones that share the same sexual ancestors but differ in ploidy may be more or less frequently recruited and successfully coexist with each other.

### Maintenance of Clonal Diversity

Clonal diversity may be determined by various non-neutral mechanisms, such as frequency-dependent selection by coevolving pathogens [52] or niche segregation among clones [53]. The maintenance of clonal diversity in asexual complexes may also exhibit a stochastic component of balance between the immigration of ‘alien’ clones and the recruitment of new clonal lineages on the one hand and their extinction and emigration on the other hand (rev. in [1], [54]). Hence, the processes affecting the clonal diversity may have much in common with the processes driving the diversity of species [55].

On the side of non-neutral processes, some sort of selection is strongly implied by the non-random spatial distribution of individual clonal lineages with respect to the parental species. No clone was found to co-occur with both parental species and all but one (MLL 85t) triploid clones co-occur with the same sperm donor species that contributed a double genomic dose to the triploid genomes (i.e. EET with *C. elongatoides* and ETT with *C. taenia*; Figure 1c). The existence of several widespread clones demonstrates that such a pattern have not have arisen by chance alone. MLL 2d, 4d, 24t, and 58t co-occur with *C. taenia* but never enter the tributaries occupied by *C. elongatoides*, although these are not isolated by any obvious obstacle for dispersal. MLL 1t has a disjunctive distribution in several tributaries co-occurring with *C. elongatoides* but it was not sampled in the main river. If we accept the delimitation of clones in hybrids with the *C. tanaitica* genome, the non-random distribution is also evident for MLL 9t, 19t–23t, and 40t (this is valid even if we consider all EEN hybrids as a single clonal lineage, since they co-occur exclusively with *C. elongatoides*).

At present, the processes that drive this type of coexistence remain unclear. Given that the genome dosage affects the morphological similarity of hybrids to one of the other parental species [28], the observed patterns may reflect interclonal selection such as the mate preferences of males for asexual females most similar to their conspecifics (i.e., the sex-mimicry mechanism; see [56]), or the species-specific habitat preference shared by the respective clones.

On the side of neutral mechanisms, we have seen that *Cobitis* clones are apparently able to disperse among distinct watersheds. Our data further indicate multiple *in situ* formation of clones, which occurs in two phases; first through multiple hybridization events producing primary diploid clones, followed by the formation of polyploid clones through the fertilization of their clonal eggs. However, Angers and Schlosser [12] showed that the discovery of several independent clones does not necessarily prove that clonal recruitment is an on-going process, or that an equilibrium between recruitment and extinction may establish. These authors documented that independent *Phoxinus eos-neogaeus* clones arose from hybridizations during the Pleistocene; however, there has been no formation of clones in recent times. Nevertheless, we demonstrated that contemporary sexual species of *Cobitis* are able to produce clonal hybrids [9] and observed microsatellite variability strongly deviates from the pattern expected under the null hypothesis of a restricted timeframe for the origin of clones (see the Results section), altogether supporting the hypothesis of continuous recruitment of *Cobitis* clones.

Of course, we must consider the possibility that sampled clones that originated recently but outside of the sampled area would appear as old, because they would possess alleles missing in local sexual populations. However, the observed distribution of age estimates significantly deviates from null expectations even when we explicitly simulated geographical structuration of parental species. This was the case even when we simulated a mixture of clones originating from sampled as well as unsampled regions interconnected by very low migration rate of 0.01 migrant per generation; supposed to be a lower limit for species cohesion [57]. Moreover, most putatively recent clones with zero *dist* values and high *I* values appeared as singletons, whereas many putatively older clones were sampled on several localities and/or in higher abundances. This pattern is consistent with the theory of the spatial distribution of alleles [58], which predicts larger ranges/abundances of older genealogical lineages. Janko et al. [1] showed that such a random-walk model might also be applicable to clonal lineages.

Overall, the recruitment of *Cobitis* clones is most likely an on-going process and their diversity and spatial distribution appears to be influenced by the influx of newly formed lineages, as well as by some type of interclonal selection.

### Implications for the Study of the Evolution of Asexuality and Polyploidy

Although investigations of mono- or polyphyly of clones with respect to their sexual ancestors is commonly applied to evaluate the dynamics of clonal recruitment, the interpretations of such information may be compromised by postformational changes overriding the original phylogenetic signal or by historical changes in recruitment rate of clones (see [12], [20] for examples). We interpret our data as strong evidence for the dynamic and continuous formation of new asexual and polyploid forms only due to the combination of fine-scale population genetics, large-scale phylogeography and unprecedented success in laboratory synthesis of clonally reproducing strains. Such detailed insights into the evolutionary history of individual asexual complexes are useful as means to test the predicted consequences of asexuality, hybridization, and polyploidy. However, we argue that drawing general hypotheses from individual case studies is very complicated, if not impossible. As exemplary contrasting cases, consider two fish complexes studied in comparable detail; *Poecilia* mollies and *Cobitis*. It is very difficult to judge the generality of the “rare formation hypothesis” of Stöck et al. [8] when considering *Poecilia*, where the initiation of asexuality was rare or even a unique event in comparison with the dynamic formation of clones and polyploids in *Cobitis* demonstrated in this paper. It remains unclear whether these strikingly differing patterns result from case-specific processes, or whether both examples represent extremes of the same process.

A possible solution to addressing the generality and the nature of the processes underlying the evolution of asexuality and polyploidy may arise from comparative studies, linking sound theoretical predictions with available phylogenetic data from independent asexual complexes. Such integration has been attempted by a limited number studies so far (e.g., [1], [11], [59]–[61]). Despite some contradictory conclusions, the age of clones and clonal diversity appeared as very promising correlates of the causal processes underlying the evolution of asexual taxa. However, these attempts have also been hampered by the application of different types of markers and methods in various case studies. Indeed, the standardization of methods for the proper delimitation of clonal lineages (and therefore the evaluation of clonal diversity; [37], [62]) and estimates of clonal ages (e.g., [63]) are issues that have been repeatedly discussed, yet remain highly actual.

Therefore, we believe that considerable effort should be invested not only in gathering new data on model case studies but also, into further developing of mutually exclusive predictions of alternative hypotheses about the evolution of asexuality and polyploidy that would allow the effective comparative or meta-analytical testing of underlying general processes.

## Supporting Information

Figure S1 **Schema of performed IBM simulation - two sexual species occur in region 1 and 2, which are interconnected by a migration rate** ***m*** **.** Interspecific hybridisation leads to the formation of clonal hybrids, which originate either in the region 1 or 2. At every 200^th^ generation, we calculate the distribution of Hartigan’s D for *dist.mut* and *dist.bp* indices. To model the effect of incomplete sampling of parental species, we further pooled the clones originating from both regions in a variable ratio and estimated the indices against first sexual demes only (see the bottom panel illustrating two such mixtures of clones).
(TIF)

Table S1 **Locality information, IDs an in** **Figure 1c** **.**
(PDF)

Table S2 **For each clone (MLL), we indicate genomes of the ancestral species, abundance, ploidy, included MLG, ancestral clone (if recognized), number of polyploid derivatives (if any), cyt** ***b*** **haplotype, and values of age indices (calculated only for individuals sampled in years 2005–2007).** (* denotes haplotypes from hybrids that were also found in Odra R. populations of parental species)
(PDF)

Table S3 **Summary of the locality ID, microsatellite data, ploidy level, genomic composition and MLG of individuals in the study.**
(PDF)

Table S4 **Allelic diversity within populations of microsatellite loci for diploid specimens.**
(PDF)

Table S5 **Microsatellite allelic diversity for diploid biotypes.**
(PDF)

Text S1 **Detailed description of methods applied to delimit the clonal lineages and to estimate the distribution of clonal ages.**
(DOC)
