# Supplementary figures and images for: Dynamic Formation of Asexual Diploid and Polyploid Lineages: Multilocus Analysis of Cobitis Reveals the Mechanisms Maintaining the Diversity of Clones
Source: PLoS One. 2012 Sep 20;7(9):e45384. doi: 10.1371/journal.pone.0045384 (PMC3447977; doi:10.1371/journal.pone.0045384)

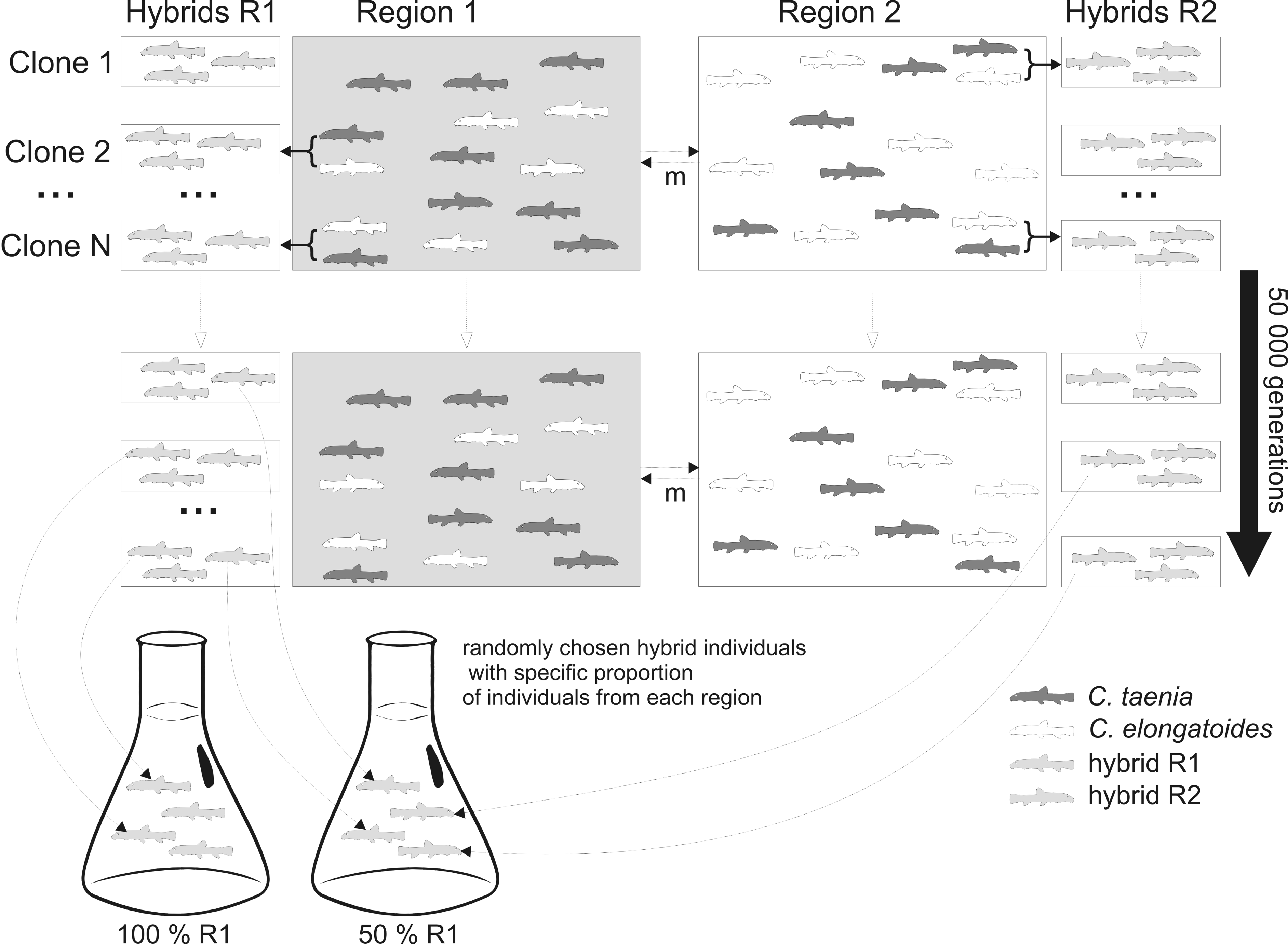

Supplement: Figure S1 — Schema of performed IBM simulation - two sexual species occur in region 1 and 2, which are interconnected by a migration rate m . Interspecific hybridisation leads to the formation of clonal hybrids, which originate either in the region 1 or 2. At every 200th generation, we calculate the distribution of Hartigan’s D for dist.mut and dist.bp indices. To model the effect of incomplete sampling of parental species, we further pooled the clones originating from both regions in a variable ratio and estimated the indices against first sexual demes only (see the bottom panel illustrating two such mixtures of clones). (TIF) [file pone.0045384.s001.tif]
