# Supplementary material for: Dynamic Formation of Asexual Diploid and Polyploid Lineages: Multilocus Analysis of Cobitis Reveals the Mechanisms Maintaining the Diversity of Clones
Source: PLoS One. 2012 Sep 20;7(9):e45384. doi: 10.1371/journal.pone.0045384 (PMC3447977; doi:10.1371/journal.pone.0045384)
Supplement: Table S1 — Locality information, IDs an in Figure 1c . (PDF) [file pone.0045384.s002.pdf]

Table S1. Locality information, IDs an in Fig. 1C.

Locality ID	River	Odra R. subbasin	Locality name	Latitude	Longitude	Date
0501	Strzałka	Widawa	Bukowa Śląska	51° 06'	17° 49'	18.4.2005
0502	Oława	Oława	Marcinkowice	50° 59'	17° 13 '	19.4.2005
0503	Smortawa	Smortawa	Janików	50° 59'	17° 22'	19.4.2005
0506	Budkowiczanka	Stobrawa	Okoły	50° 52'	17° 56'	20.4.2005
0507	Budkowiczanka	Stobrawa	Zagwiździe	50° 52 '	18° 02'	22.4.2005
0508	Dziewicza Struga	Kaczawa	Szczytniki Małe	51° 15'	16° 16 '	21.4.2005
0509	Wierzbiak	Kaczawa	Kunice	51° 14'	16° 14'	21.4.2005
0510	Skora	Kaczawa	Grzymalin	51° 17'	16° 05'	21.4.2005
0511	Sumina	Ruda	Sumina	50° 09'	18° 24'	22.4.2005
0513	Ślesiński Canal	Warta	Żółwiniec	52° 25'	18° 21'	29.9.2005
0515	Odra	-	Frankfurt am Oder	52° 20'	14° 33'	17.11.2005
0601	Odra	-	Czerna	51° 43'	15° 54'	4.9.2006
0602	Czarna	Rudna	Czerńczyce	51° 37'	16° 13'	4.9.2006
0603	Zimnica	direct Odra basin	Niemstów	51° 22'	16° 17'	5.9.2006
0604	Krzycki Rów	direct Odra basin	Kierzno	51° 45'	15° 55'	5.9.2006
0606	Ołobok Canal	direct Odra basin	Nietkowice	52° 04'	15° 22'	6.9.2006
0607	Krzesiński Canal	direct Odra basin	Krzesin	52° 05'	14° 45'	7.9.2006
0608	Odra	-	Czarnowo	52° 03'	14° 57'	7.9.2006
0701	Barycz	Barycz	Ryczeń	51° 36'	16° 30'	13.6.2007
0702	Rudna	Rudna	Retków	51° 36'	16° 12'	14.6.2007
0703	Rudna	Rudna	Głogów	51° 38'	16° 09'	14.6.2007
0705	Odra	-	Wietszyce	51° 38'	16° 18'	15.6.2007
0706	Odra	-	Ciechłowice	51° 29'	16° 27'	10.8.2007
0801	Polska Woda	Barycz	Wielgie Milickie	51° 31'	17° 30'	22.9.2008
0804	Widawa	Widawa	Kiełczów	51° 07'	17° 10'	24.9.2008
0901	Odra	-	Kostrzyn	25° 36'	14° 36'	24.4.2009
0903	Odra	-	Ratowice	51° 01'	17° 16'	24.8.2009
