# Supplementary material for: Dynamic Formation of Asexual Diploid and Polyploid Lineages: Multilocus Analysis of Cobitis Reveals the Mechanisms Maintaining the Diversity of Clones
Source: PLoS One. 2012 Sep 20;7(9):e45384. doi: 10.1371/journal.pone.0045384 (PMC3447977; doi:10.1371/journal.pone.0045384)
Supplement: Table S2 — For each clone (MLL), we indicate genomes of the ancestral species, abundance, ploidy, included MLG, ancestral clone (if recognized), number of polyploid derivatives (if any), cyt b haplotype, and values of age indices (calculated only for individuals sampled in years 2005–2007). (* denotes haplotypes from hybrids that were also found in Odra R. populations of parental species) (PDF) [file pone.0045384.s003.pdf]

Table S2. For each clone (MLL), we indicate genomes of the ancestral species, abundance, ploidy, included MLG, ancestral clone (if recognized), number of polyploid derivatives (if any), cyt b haplotype, and values of age indices (calculated only for individuals sampled in years 2005-2007). (* denotes haplotypes from hybrids that were also found in Odra R. populations of parental species)

MLL	Genomes of parental species	Number of specimens	Ploidy	MLGs included in MLL (if more than one)	Ancestral (lower ploidy) MLL	Number of polyploid derivatives	Cytochrome *b* haplotype	*dist.mut*	*dist.bp*	Tomiuk & Loeschcke's *I* (s.e.)
2d	E, T	61	2n	*2d,3d,11d,13d,14d,15d,17d*	NA	23	1-T1*	0.7	1.4	0.704 (0.026)
8d	E, T	3	2n	*8d,9d*	NA	2	1-T1*	1.7	5.3	0.671 (0.029)
1d	E, T	1	2n		NA	0	1-T1*	2	10	0.66 (0.03)
4d	E, T	2	2n		NA	1	NA	2	12	0.635 (0.034)
5d	E, T	1	2n		NA	0	E8*	1	1	0.681 (0.029)
6d	E, T	1	2n		NA	2	T14	2	5	0.662 (0.03)
7d	E, T	1	2n		NA	0	35	1	1	0.678 (0.03)
10d	E, T	13	2n		NA	2	1-T1*	1	6	0.601 (0.031)
12d	E, T	3	2n		NA	2	1-T1*	1	8	0.628 (0.034)
18d	E, T	1	2n		NA	0	E8*	1	8	0.683 (0.031)
19d	E, T	1	2n		NA	2	1-T1*	0	0	0.655 (0.03)
4t	E, T, N	10	3n	*4t,25t,80t*	NA	3	T17*, T18	2.6667	20.6667	0.698 (0.023)
71t	E, T, N	2	3n	*71t,73t*	NA	0	T17*, 2-T9*	3	21	0.704 (0.021)
44t	E, T, N	10	3n	*44t,48t,81t,84t*	NA	1	T12*, T25*, 1-T1*, 2-T9*	3.3333	26	0.695 (0.019)
9t	E, N	23	3n	*9t,13t,17t,99t*	NA	7	E39, E38	5.3333	32.8667	0.665 (0.026)
1t	E, T	22	3n	*1t,2t,8t,7t,12t*	NA	6	NA	0.0333	0.0333	0.704 (0.026)
19t	E, T, N	23	3n	*19t,23t,50t,52t,59t,74t,75t*	NA	2	T27	2.6667	21.4667	0.707 (0.021)
58t	E, T	7	3n	*58t,64t,82t*	NA	0	2-T9*	4	24.6667	0.7 (0.023)
21t	E, T, N	3	3n	*21t,27t*	NA	0	2-T9*, T18	1.3333	18.6667	0.735 (0.021)
20t	E, T, N	5	3n	*20t,76t*	NA	0	T27	1.4667	19.4667	0.673 (0.025)
46t	E, T	2	3n	*46t,47t*	NA	0	1-T1*	2.6667	24	0.67 (0.022)
91t	E, T	6	3n	*91t,94t*	NA	1	NA	NA	NA	0.67 (0.03)
16t	E, T	3	3n	*16t,101t*	NA	0	1-T1*	NA	NA	0.703 (0.026)
92t	E, T	2	3n	*92t, 95t*	NA	0	NA	NA	NA	NA
3t	E, T	1	3n		NA	0	T11	0	0	0.743 (0.022)
5t	E, T, N	1	3n		NA	0	NA	2.6667	23.3333	0.685 (0.024)
6t	E, T	2	3n		NA	0	NA	4	24.6667	0.686 (0.025)
10t	E, N	1	3n		NA	0	E38	5.3333	34	0.521 (0.041)
11t	E, T	1	3n		NA	0	1-T1*	0.6667	0.6667	0.689 (0.028)
14t	E, N	1	3n		NA	0	E38	4.6667	31.3333	0.559 (0.04)

15t	E, T, N	1	3n	NA	0	2-T9*	2.6667	20.6667	0.7 (0.022)
18t	E, T	1	3n	NA	0	1-T1*	0	0	0.756 (0.022)
22t	E, T	1	3n	2d	0	1-T1*	0	0	0.81 (0.019)
24t	E, T	2	3n	2d	1	1-T1*	0.6667	2.6667	0.733 (0.023)
26t	E, T	1	3n	NA	0	NA	3.3333	24	0.633 (0.029)
28t	E, T	1	3n	NA	0	1-T1*	0	0	0.782 (0.02)
29t	E, T	1	3n	NA	0	1-T1*	0.6667	1.3333	0.789 (0.024)
30t	E, T	1	3n	2d	0	1-T1*	0.6667	1.3333	0.762 (0.023)
31t	E, T	1	3n	NA	0	1-T1*	0	0	0.792 (0.021)
32t	E, T	1	3n	2d	0	1-T1*	0	0	0.795 (0.02)
33t	E, T	1	3n	2d	0	1-T1*	0	0	0.799 (0.021)
34t	E, T	1	3n	2d	0	1-T1*	0	0	0.771 (0.021)
35t	E, T	1	3n	2d	0	1-T1*	0.6667	1.3333	0.808 (0.018)
36t	E, T	1	3n	2d	0	1-T1*	0	0	0.8 (0.02)
37t	E, T	1	3n	2d	0	1-T1*	0	0	0.789 (0.02)
38t	E, T	1	3n	4d	0	T15	0	0	0.736 (0.021)
39t	E, T	1	3n	8d	0	1-T1*	0	0	0.744 (0.021)
40t	E, T, N	3	3n	NA	0	T24	3.3333	24.6667	0.711 (0.023)
41t	E, T	1	3n	6d	0	1-T1*	0.6667	0.6667	0.732 (0.023)
42t	E, T	1	3n	8d	0	1-T1*	0	0	0.798 (0.019)
43t	E, T	1	3n	6d	0	1-T1*	0	0	0.787 (0.016)
45t	E, T	1	3n	NA	0	1-T1*	0	0	0.791 (0.021)
49t	E, T, N	1	3n	NA	0	T17*	3.3333	25.3333	0.684 (0.024)
51t	E, T, N	2	3n	NA	0	T27	2.6667	21.3333	0.683 (0.023)
53t	E, T	1	3n	10d	0	1-T1*	0.6667	1.3333	0.776 (0.017)
54t	E, T	1	3n	12d	0	1-T1*	0	0	0.749 (0.02)
55t	E, T	1	3n	NA	0	45	3.3333	24	0.629 (0.029)
56t	E, T	1	3n	2d	0	1-T1*	0	0	0.789 (0.019)
57t	E, T	1	3n	2d	0	1-T1*	0	0	0.768 (0.019)
60t	E, T, N	1	3n	NA	0	2-T9*	2	20	0.692 (0.019)
61t	E, T	1	3n	2d	0	1-T1*	0	0	0.766 (0.019)
62t	E, T	1	3n	NA	0	T13	0	0	0.753 (0.02)
63t	E, T	1	3n	NA	0	1-T1*	0	0	0.768 (0.022)
65t	E, T	1	3n	2d	0	1-T1*	0	0	0.831 (0.017)
66t	E, T	1	3n	2d	0	1-T1*	0	0	0.83 (0.019)
67t	E, T	1	3n	10d	0	1-T1*	0	0	0.72 (0.023)
68t	E, T	1	3n	2d	0	1-T1*	0	0	0.789 (0.02)
69t	E, T	1	3n	2d	0	1-T1*	0	0	0.817 (0.018)
70t	E, T	1	3n	2d	0	1-T1*	0.6667	1.3333	0.776 (0.019)

72t	E, T	1	3n	2d	0	1-T1*	0.6667	1.3333	0.828 (0.017)
77t	E, T	1	3n	2d	0	1-T1*	0	0	0.764 (0.019)
78t	E, T	1	3n	2d	0	1-T1*	0	0	0.793 (0.017)
79t	E, T	1	3n	NA	0	1-T1*	0	0	0.716 (0.023)
83t	E, T	1	3n	NA	0	2-T9*	2.6667	20.6667	0.682 (0.025)
85t	E, T	1	3n	19d	0	1-T1*	0	0	0.687 (0.029)
86t	E, T	1	3n	NA	0	7	4	24.6667	0.687 (0.025)
87t	E, T	1	3n	12d	0	1-T1*	0	0	0.768 (0.02)
88t	E, T	7	3n	NA	2	NA	NA	NA	NA
89t	E, T	2	3n	NA	0	NA	NA	NA	NA
90t	E, T	1	3n	NA	0	NA	NA	NA	NA
93t	E, T	3	3n	NA	0	NA	NA	NA	NA
96t	E, T	3	3n	NA	1	NA	NA	NA	NA
97t	E, T	1	3n	NA	0	NA	NA	NA	NA
98t	E, T	1	3n	NA	0	NA	NA	NA	NA
100t	E, T	1	3n	NA	0	NA	NA	NA	NA
1te	E, T	1	4n	NA	0	NA	0.5	0.5	NA
2te	E, T	1	4n	1	0	NA	0	0	NA
3te	E, T, N	1	4n	4	0	NA	0	0	NA
4te	E, T, N	1	4n	NA	0	NA	0	0	NA
5te	E, T	1	4n	1	0	NA	0	0	NA
6te	E, T	1	4n	1	0	NA	0	0	NA
7te	E, T	1	4n	1	0	NA	0	0	NA
8te	E, T, N	1	4n	NA	0	NA	0	0	NA
9te	E, T	1	4n	NA	0	NA	0	0	NA
10te	E, N	1	4n	9	0	NA	0	0	NA
11te	E, N	1	4n	NA	0	NA	0	0	NA
12te	E, N	1	4n	9	0	NA	0	0	NA
13te	E, N	1	4n	9	0	NA	0	0	NA
14te	E, T	1	4n	1	0	NA	0	0	NA
15te	E, N	1	4n	9	0	NA	0	0	NA
16te	E, T, N	1	4n	19	0	NA	0	0	NA
17te	E, T, N	1	4n	NA	0	NA	0	0	NA
18te	E, T, N	1	4n	NA	0	NA	0	0	NA
19te	E, T, N	1	4n	NA	0	NA	0	0	NA
20te	E, T, N	1	4n	24	0	NA	0.5	1	NA
21te	E, T, N	1	4n	NA	0	NA	0.5	1	NA
22te	E, T, N	1	4n	23	0	NA	0	0	NA
23te	E, T, N	1	4n	NA	0	NA	0.5	0.5	NA

24te	E, T, N	1	4n	NA	0	NA	0	0	NA
25te	E, T, N	1	4n	84	0	NA	0	0	NA
26te	E, T	1	4n	1	0	NA	NA	NA	NA
27te	E, N	1	4n	9	0	NA	NA	NA	NA
28te	E, T	1	4n	88	0	NA	NA	NA	NA
29te	E, N	1	4n	NA	0	NA	NA	NA	NA
30te	E, N	1	4n	NA	0	NA	NA	NA	NA
31te	E, T	1	4n	88	0	NA	NA	NA	NA
32te	E, N	1	4n	9	0	NA	NA	NA	NA
33te	E, T	1	4n	91	0	NA	NA	NA	NA
34te	E, N	1	4n	17	0	NA	NA	NA	NA
35te	E, T	1	4n	96	0	NA	NA	NA	NA
36te	E, N	1	4n	NA	0	NA	NA	NA	NA
37te	E, T, N	1	4n	NA	0	NA	NA	NA	NA
38te	E, T, N	1	4n	4	0	NA	NA	NA	NA
39te	E, N	1	4n	4	0	NA	NA	NA	NA
