# Supplementary material for: Dynamic Formation of Asexual Diploid and Polyploid Lineages: Multilocus Analysis of Cobitis Reveals the Mechanisms Maintaining the Diversity of Clones
Source: PLoS One. 2012 Sep 20;7(9):e45384. doi: 10.1371/journal.pone.0045384 (PMC3447977; doi:10.1371/journal.pone.0045384)
Supplement: Table S3 — Summary of the locality ID, microsatellite data, ploidy level, genomic composition and MLG of individuals in the study. (PDF) [file pone.0045384.s004.pdf]

Table S3. Summary of the locality ID (like in Fig. 1 c), microsatellite data, ploidy level, genomic composition and MLGs of individuals in the study; (* - uncertain genom qantity).

Sample name	Locality ID	Microsatellite locus										Ploidy	Genomic composition	MLG
		cota_006	cota_010	cota_027	cota_032	cota_033	cota_037	cota_041	cota_068	cota_093	cota_111			
HZ050102	0501	313351353	138170188	302302	212232	211223	277296	330376	199211	303311345	218220258	3n	EET	*1t*
HZ050104	0501	313351353	138170188	302302	212232	211223	277296	330376	199211	303311345	218220258	3n	EET	*1t*
HZ050103	0501	313351353	138170188	302302	212232	211223	277280296	330376	199211	303311345	218220258	3n	EET	*2t*
HZ05015	0501	353357	138164170188	302302	212232	223223	263271	330330	199211	303311313345	218220258	4n	EEET	*1te*
HZ050105	0501	313342351353	164178	n.a.	212212	211223	276277296	330330	211211	309313	218218	4n	EEET	*2te*
HZ05021	0502	313351	138188	307307	212232	211235	277296	330376	199211	311345	220250	2n	ET	*1d*
HZ05024	0502	335339	138138	307307	265269	211211	272277	374382	199199	333357	250250	2n	TT	
HZ05022	0502	348351357	138178	307307	212232269	211223	267277283	330374376	199211	311337345	220250258	3n	ETT	*3t*
HZ05023	0502	346351359	138180	287287	212232	211231	272280326	330366378	199215	307335343	218252268	3n	ETN	*4t*
HZ05037	0503	348348	138138	307307	269269	211231	272272	370378	199199	333335	250264	2n	TT	
HZ05031	0503	346351359	138180	287287	212232	211231	272280326	330366378	199215	307335343	218252268	3n	ETN	*4t*
HZ05032	0503	346351359	138180	287287	212232	211231	272280326	330366378	199215	307335343	218252268	3n	ETN	*4t*
HZ050310	0503	346351367	138180	287287	212232	211231	272280330	330366378	199215	307335343	218256268	3n	ETN	*5t*
HZ05035	0503	309351	138186	287307	212269277	211223	284301326	330366374	199215	307315337	218252268	3n	ETT	*6t*
HZ05036	0503	309351	138186	287307	212269277	211223	284301326	330366374	199215	307315337	218252268	3n	ETT	*6t*
HZ050311	0503	346351359	138180	287307	212232	211231	268272280326	330366370378	199215	307335343353	218252268	4n	ETTN	*3te*
HZ05038	0503	346355359	138180	287312	212232269	211231	272276280326	330366370378	199215	307335343353	218252268	4n	ETTN	*4te*
HZ05061	0506	313353	138170188	302302	212232	211223	277296	330376	199211	303311345	218220258	3n	EET	*7t*
HZ050716	0507	342376	188188	000000	212212	223239	275275	330330	211211	303303	218218	2n	EE	
HZ050717	0507	353376	178188	000000	212212	239239	261296	330330	211211	303309	218218	2n	EE	
HZ05072	0507	353376	170176	000000	212212	239239	267296	330330	211211	303303	216218	2n	EE	
HZ05073	0507	376376	178188	000000	212212	223239	296296	330330	211211	303309	218220	2n	EE	
HZ05074	0507	353361	164164	000000	212212	223223	263274	330330	211211	303311	218220	2n	EE	
HZ05075	0507	353353	178188	000000	212212	239239	263296	330330	211211	303303	216218	2n	EE	
HZ05076	0507	353353	164178	000000	212212	223239	275296	330330	211211	309311	218218	2n	EE	
HZ05077	0507	342353	178188	000000	212212	223239	263296	330330	211211	303303	218218	2n	EE	
HZ05078	0507	357357	164178	000000	212212	223239	263296	330330	211211	303309	218218	2n	EE	
HZ05071	0507	313351353	138170188	302302	212232	211223	277296	330376	199211	303311345	218220258	3n	EET	*1t*
HZ050713	0507	313351353	138170188	302302	212232	211223	277296	330376	199211	303311345	218220258	3n	EET	*1t*
HZ050714	0507	313351353	138170188	302302	212232	211223	277296	330376	199211	303311345	218220258	3n	EET	*1t*
HZ050710	0507	313353	138170188	302302	212232	211223	277296	330376	199211	303311345	218220258	3n	EET	*7t*

HZ050711	0507	313353355	138170188	302302	212232	211223	277296	330376	199211	303311345	218220258	3n	EET	*8t*
HZ050715	0507	309346351	138180186	287287	212232	211223231	280284326	330366	215219	303307315	218268	3n	EEN	*9t*
HZ050712	0507	313351353357	138170178188	302302	212232	211223239	267277296	330376	199211	303311345	218220258	4n	EEET	*5te*
HZ050718	0507	313351353	138170188	302302	212232	211223	277296	330376	199211	303311345	218220258	4n	EEET	*6te*
HZ05079	0507	313351353357	138164170188	302302	212232	211223	275277296	330332376	199211	303311345	218220258	4n	EEET	*7te*
RQ107	0507	313351353	138170188	302302	212232	211223239	n.a.	330376	199211	303311345	218220258	3n	EET	*101t*
RQ121	0507	313351353	138170188	302302	212232	211223239	n.a.	330376	199211	303311345	218220258	3n	EET	*101t*
HZ05081	0508	353353	158164	000000	212212	223239	275275	330330	211211	303311	218218	2n	EE	
HZ05082	0508	353373	164176	000000	212212	223239	275296	330330	211211	303309	216220	2n	EE	
HZ05083	0508	357357	178178	000000	212212	223239	265300	330330	211211	303305	216216	2n	EE	
HZ05084	0508	353353	164178	000000	212212	239239	263300	332332	211211	303309	216216	2n	EE	
HZ05085	0508	353380	178188	000000	212212	223223	265284	330332	211211	303311	216220	2n	EE	
HZ05086	0508	342353	170178	000000	212212	223239	275296	330330	211211	309309	218220	2n	EE	
HZ050910	0509	342357	164178	000000	212212	239239	261296	330330	211211	303311	216218	2n	EE	
HZ050911	0509	353353	160178	000000	212212	239239	261267	330330	211211	309311	216216	2n	EE	
HZ050912	0509	353353	164188	000000	212212	223223	275284	330330	211211	309313	216218	2n	EE	
HZ05094	0509	353353	160178	000000	212212	239239	267302	330330	211211	303309	218218	2n	EE	
HZ05095	0509	353380	178188	000000	212212	239239	267274	330330	211211	303309	216218	2n	EE	
HZ05097	0509	353365	164188	000000	212212	223239	261300	330330	211211	303309	218220	2n	EE	
HZ05099	0509	357361	178178	000000	212212	223239	275284	330332	211211	303311	218218	2n	EE	
HZ05091	0509	313351353	138170188	302302	212232	211223	277296	330376	199211	303311345	218220258	3n	EET	*1t*
HZ05093	0509	309346351	138180186	287287	212232	211223231	280284326	330366	215219	303307315	218268	3n	EEN	*9t*
HZ050913	0509	309344350	138180186	287287	212232	211223231	280284326	330366	215219	303307313	218278	3n	EEN	*10t*
HZ05096	0509	313353	138170188	302302	212232	211223	277286296	330376	199211	303311345	218220258	3n	EET	*11t*
HZ05092	0509	309346351357	138180186	287287	212232	211223231239	261280284326	330332366	211215219	307309315	218268	4n	EENN*	*8te*
HZ05098	0509	313353	138170178188	302302	212232	n.a.	n.a.	n.a.	199211	303311345	218220262	4n	EEET*	*9te*
HZ051023	0510	313351353	138170188	302302	212232	211223	277296	330376	199211	303311345	218220258	3n	EET	*1t*
HZ05106	0510	313351353	138170188	302302	212232	211223	277296	330376	199211	303311345	218220258	3n	EET	*1t*
HZ05102	0510	313353	138170188	302302	212232	211223	277296	330376	199211	303311345	218220258	3n	EET	*7t*
HZ05101	0510	313351353	138170188	302302	212232	211223	277296	330376	199211	303311345	218220262	3n	EET	*12t*
HZ051011	0510	313351353	138170188	302302	212232	211223	277296	330376	199211	303311345	218220262	3n	EET	*12t*
HZ051015	0510	313351353	138170188	302302	212232	211223	277296	330376	199211	303311345	218220262	3n	EET	*12t*
HZ051017	0510	313351353	138170188	302302	212232	211223	277296	330376	199211	303311345	218220262	3n	EET	*12t*
HZ051020	0510	313351353	138170188	302302	212232	211223	277296	330376	199211	303311345	218220262	3n	EET	*12t*
HZ05104	0510	313351353	138170188	302302	212232	211223	277296	330376	199211	303311345	218220262	3n	EET	*12t*
HZ051012	0510	309346351	138180186	287287	212232	211223231	280284326	330366	215219	303307315	218268	3n	EEN	*9t*

HZ051018	0510	309346351	138180186	287287	212232	211223231	280284326	330366	215219	303307315	218268	3n	EEN	*9t*
HZ051022	0510	309346351	138180186	287287	212232	211223231	280284326	330366	215219	303307315	218268	3n	EEN	*9t*
HZ051025	0510	309346351	138180186	287287	212232	211223231	280284326	330366	215219	303307315	218268	3n	EEN	*9t*
HZ05105	0510	309346351	138180186	287287	212232	211223231	280284326	330366	215219	303307315	218268	3n	EEN	*9t*
HZ05108	0510	309346351	138180186	287287	212232	211223231	280284326	330366	215219	303307315	218268	3n	EEN	*9t*
HZ051013	0510	309346348	138180186	287287	212232	211223231	280284326	330366	215219	303307315	218268	3n	EEN	*13t*
HZ051016	0510	309344350	138180186	287307	212232	211223231	280284326	330366	215219	303307313	218268	3n	EEN	*14t*
HZ051021	0510	336346351	138180	307307	212232	211231	272280326	330366374	199215	307341	218252	3n	ETN	*15t*
HZ05103	0510	313353	138170188	302302	212232	211223239	277296	330376	199211	303311345	218220258	3n	EET	*16t*
HZ051010	0510	309342346351	138164180186	287287	212232	211223231239	267280284326	330366	211215219	303307309315	218220268	4n	EEEN	*10te*
HZ051014	0510	309346351376	138164180186	287287	212232	211223231239	274280284330	330366	211215219	303305307315	218268	4n	EEEN	*11te*
HZ051019	0510	309346351369	138180186	287287	212232	211223231239	280284326	330366	211215219	303307315	218268	4n	EEEN*	*12te*
HZ051024	0510	309346351373	n.a.	n.a.	n.a.	211223231	265280284326	330366	n.a.	n.a.	n.a.	4n	EEEN*	*13te*
HZ05109	0510	313342351353	138170188	302302	212232	211223239	277296	330376	199211	303309311345	218220258	4n	EEET	*14te*
HZ051102	0511	342353	170176	000000	212212	223239	267296	330330	211211	303311	218218	2n	EE	
HZ051103	0511	350353	164178	000000	212212	223223	267275	330330	211211	303311	218218	2n	EE	
HZ051104	0511	357357	178180	000000	212212	239239	261275	330330	211211	303303	218218	2n	EE	
HZ051110	0511	353357	178188	000000	212212	239239	296296	330332	211211	303303	218218	2n	EE	
HZ051113	0511	346350	158176	000000	212212	223223	275296	330330	211211	303303	218218	2n	EE	
HZ051114	0511	346357	164176	000000	212212	223239	275296	330330	211211	303303	218220	2n	EE	
HZ051101	0511	309346351	138180186	287287	212232	211223231	280284326	330366	215219	303307315	218268	3n	EEN	*9t*
HZ051105	0511	309346351	138180186	287287	212232	211223231	280284326	330366	215219	303307315	218268	3n	EEN	*9t*
HZ051108	0511	309346351	138180186	287287	212232	211223231	280284326	330366	215219	303307315	218268	3n	EEN	*9t*
HZ051109	0511	309346351	138180186	287287	212232	211223231	280284326	330366	215219	303307315	218268	3n	EEN	*9t*
HZ051111	0511	309346351	138180186	287287	212232	211223231	280284326	330366	215219	303307315	218268	3n	EEN	*9t*
HZ051106	0511	309346351	138180186	287287	212232	211223231	280284328	330366	215219	303307315	218268	3n	EEN	*17t*
HZ051107	0511	309346351	138178180186	287287	212232	211223231	280284326	330366	215219	303307315	218268	4n	EEEN	*15te*
HZ051310	0513	355355	138138	287292	232269	211231	272274	376378	199199	329353	250266	2n	TT	
HZ051311	0513	332363	138138	287302	232269	211231	274276	378386	199199	341353	252254	2n	TT	
HZ051312	0513	351363	138138	287287	232269	231231	272274	374386	199199	337353	252258	2n	TT	
HZ051313	0513	348355	138138	287312	232269	211231	276276	370370	199199	333337	258264	2n	TT	
HZ051314	0513	328355	138138	287297	265269	231231	276279	374378	199199	329353	264266	2n	TT	
HZ051315	0513	359400	138138	292307	273277	211211	272274	374374	199199	341353	260266	2n	TT	
HZ051316	0513	355359	138138	287302	232269	211231	276277	360370	199199	341345	246252	2n	TT	
HZ051318	0513	344344	138138	287287	232232	231231	272283	358374	199199	337349	246266	2n	TT	
HZ051319	0513	338346	138138	287307	232232	211231	274276	378378	199199	339351	250260	2n	TT	

HZ05132	0513	348355	138138	287287	232232	211231	274276	378378	199199	331337	250254	2n	TT	
HZ051320	0513	328340	138138	287307	232269	211231	272283	374378	199199	335349	254254	2n	TT	
HZ051321	0513	332344	138138	287287	256269	211211	274283	374380	199199	341353	262264	2n	TT	
HZ051322	0513	348363	138138	287287	232273	231231	274291	376384	199199	329333	258262	2n	TT	
HZ051323	0513	348355	138138	287287	232232	211231	272272	374378	199199	337341	248260	2n	TT	
HZ051324	0513	348348	138138	287287	232232	211231	276283	376380	199199	331337	258264	2n	TT	
HZ051325	0513	363363	138138	287287	232269	211231	268276	364380	199199	333349	250250	2n	TT	
HZ051326	0513	355363	138138	287302	232232	211211	276279	370370	199199	337341	260266	2n	TT	
HZ051327	0513	351363	138138	287307	232232	231231	274276	374378	199199	333333	252264	2n	TT	
HZ051328	0513	328355	138138	287287	232232	211231	274276	378386	199199	333333	250250	2n	TT	
HZ051329	0513	328340	138138	287307	232269	231231	277279	374384	199199	325349	246264	2n	TT	
HZ05133	0513	339359	138138	287297	232269	231231	274276	374374	199199	337341	258264	2n	TT	
HZ051330	0513	328332	138138	287287	232232	211231	272276	374378	199199	341349	250266	2n	TT	
HZ051331	0513	332344	138138	287292	232269	211231	274276	370382	199199	333341	250254	2n	TT	
HZ051332	0513	351359	138138	287287	232269	211231	272279	380382	199199	339349	250250	2n	TT	
HZ051333	0513	336363	138138	287287	232232	211231	272276	374380	199199	333353	258264	2n	TT	
HZ051334	0513	336336	138138	287302	232232	231231	270274	384384	199199	333347	250252	2n	TT	
HZ051336	0513	340359	138138	287287	232232	211211	272276	374382	199199	333349	248250	2n	TT	
HZ051337	0513	348382	138138	287287	232273	211211	272274	378382	199199	353353	250258	2n	TT	
HZ05134	0513	344351	138138	287312	232232	211211	272272	374374	199199	337345	250252	2n	TT	
HZ05135	0513	319331	138138	292302	232269	211231	272276	378380	199199	341343	250252	2n	TT	
HZ05136	0513	348351	138138	287287	232232	211231	272276	380386	199199	337353	252262	2n	TT	
HZ05137	0513	320336	138138	287312	232232	211231	272276	374380	199199	333341	258262	2n	TT	
HZ05138	0513	336398	138138	287302	232232	211231	274276	378382	199199	337349	250260	2n	TT	
HZ05131	0513	313351	138188	287307	212232	211223	272274296	330364376	199211	311333345	218250258	3n	ETT	*18t*
HZ05154	0515	351369	138164	287287	212232	211223	272296	330376	199211	311341	218252	2n	ET	*2d*
HZ05157	0515	336340	138138	307317	232273	211211	279285	370374	199199	347353	252268	2n	TT	
HZ05158	0515	340367	138138	307312	232232	211211	272272	374378	199199	339357	250252	2n	TT	
HZ05151	0515	346355382	138180	287302	212232	211223	272284328	330366374	199215	307315353	218252268	3n	ETN	*19t*
HZ05152	0515	340350380	138180	287302	212232	211223	272284326	330366374	199216	305311347	218254270	3n	ETN	*20t*
HZ05156	0515	340350380	138180	287302	212232	211223	272284326	330366374	199216	305311347	218254270	3n	ETN	*20t*
HZ05153	0515	344346351	138180	287312	212232273	211231	276284326	330366378	199215	303335345	218252268	3n	ETN	*21t*
HZ05159	0515	351359369	138164	287307	212232	211223	272296	330376378	199211	311329341	218250252	3n	ETT	*22t*
HZ05155	0515	336346355382	138180	287302307	212232265	211223	272274284328	330366374376	199215	307315333353	218250252268	4n	ETTN*	*16te*
HZ51510	0515	346351371	138180	287307	212232273	211231	277280326	330366378	199219	307315347	218250260	3n	ETN	*40t*
HZ06011	0601	346355382	138180	287302	212232	211223	272284326	330366374	199215	307315353	218252268	3n	ETN	*23t*

HZ07041	0601	351369	138164	287287	212232	211223	272296	330376	199211	311341	218252	2n	ET	*2d*
HZ070411	0601	351369	138164	287287	212232	211223	272296	330376	199211	311341	218252	2n	ET	*2d*
HZ070412	0601	351369	138164	287287	212232	211223	272296	330376	199211	311341	218252	2n	ET	*2d*
HZ070413	0601	351369	138164	287287	212232	211223	272296	330376	199211	311341	218252	2n	ET	*2d*
HZ070415	0601	351369	138164	287287	212232	211223	272296	330376	199211	311341	218252	2n	ET	*2d*
HZ070422	0601	351369	138164	287287	212232	211223	272296	330376	199211	311341	218252	2n	ET	*2d*
HZ070423	0601	351369	138164	287287	212232	211223	272296	330376	199211	311341	218252	2n	ET	*2d*
HZ070427	0601	351369	138164	287287	212232	211223	272296	330376	199211	311341	218252	2n	ET	*2d*
HZ070431	0601	351369	138164	287287	212232	211223	272296	330376	199211	311341	218252	2n	ET	*2d*
HZ070434	0601	351369	138164	287287	212232	211223	272296	330376	199211	311341	218252	2n	ET	*2d*
HZ070436	0601	351369	138164	287287	212232	211223	272296	330376	199211	311341	218252	2n	ET	*2d*
HZ070437	0601	351369	138164	287287	212232	211223	272296	330376	199211	311341	218252	2n	ET	*2d*
HZ070442	0601	351369	138164	287287	212232	211223	272296	330376	199211	311341	218252	2n	ET	*2d*
HZ070443	0601	351369	138164	287287	212232	211223	272296	330376	199211	311341	218252	2n	ET	*2d*
HZ070445	0601	351369	138164	287287	212232	211223	272296	330376	199211	311341	218252	2n	ET	*2d*
HZ070449	0601	351369	138164	287287	212232	211223	272296	330376	199211	311341	218252	2n	ET	*2d*
HZ07045	0601	351369	138164	287287	212232	211223	272296	330376	199211	311341	218252	2n	ET	*2d*
HZ070450	0601	351369	138164	287287	212232	211223	272296	330376	199211	311341	218252	2n	ET	*2d*
HZ07046	0601	351369	138164	287287	212232	211223	272296	330376	199211	311341	218252	2n	ET	*2d*
HZ07048	0601	351369	138164	287287	212232	211223	272296	330376	199211	311341	218252	2n	ET	*2d*
HZ07044	0601	351373	138164	287287	212232	211223	272296	330376	199211	311341	218252	2n	ET	*13d*
HZ070447	0601	351373	138164	287287	212232	211223	272296	330376	199211	311341	218252	2n	ET	*13d*
HZ070416	0601	350367	138164	287287	212232	211223	272296	330376	199211	311341	218252	2n	ET	*14d*
HZ070419	0601	355369	138164	287287	212232	211223	272296	330376	199211	311341	218252	2n	ET	*15d*
HZ070435	0601	351369	138164	287287	212232	211223	268296	330376	199211	311341	218252	2n	ET	*17d*
HZ070433	0601	340340	138138	307307	232269	211231	272?	370374	199199	347353	250256	2n	TT	
HZ070439	0601	346355382	138180	287302	212232	211223	272284326	330366374	199215	307315353	218252268	3n	ETN	*23t*
HZ070441	0601	346355	138180	287302	212232	211223	272284326	330366374	199215	307315353	218252268	3n	ETN	*51t*
HZ07042	0601	309340351	138186	287307	212269277	211223	284297326	330366374	199215	307315341	218252268	3n	ETT	*64t*
HZ070414	0601	340351369	138164	287287	212232	211223	272284296	330376378	199211	311341	218250252	3n	ETT	*61t*
HZ070417	0601	344351353	138188	307312	212232273	211239	276284296	330374376	199211	303333341	218250270	3n	ETT	*62t*
HZ070418	0601	340369	138164	287307	212232269	211223	272296	330376	199211	311341343	218250252	3n	ETT	*63t*
HZ070426	0601	332351369	138164	287307	212232	211223	272277296	330374376	199211	311341353	218252	3n	ETT	*65t*
HZ070429	0601	340351369	138164	287307	212232	211223231	272296	330374376	199211	311341343	218252272	3n	ETT	*66t*
HZ07043	0601	313332351	138170	307307	212269	211223231	277283296	330374376	199211	303341	218250252	3n	ETT	*67t*
HZ070438	0601	340351369	138164	287312	212232	211223	272283296	330376380	199211	311341	218250252	3n	ETT	*68t*

HZ070440	0601	351369	138164	287307	212232	211223231	272277296	330374376	199211	311341345	218250252	3n	ETT	*69t*
HZ070446	0601	348351369	138164	287307	212232	211223	272287296	330370376	199211	311341345	218252268	3n	ETT	*70t*
HZ070451	0601	346351	138180	287287	212232	211231	272280326	330366378	199215	307336343	218252268	3n	ETN	*71t*
HZ07047	0601	344351369	138164	287307	212232	211223235	272289296	330374376	199211	311333341	218250252	3n	ETT	*72t*
HZ070420	0601	344346359363	138180	287307	212232	n.a.	n.a.	n.a.	199215	307335343353	218252264268	4n	ETTN*	*17te*
HZ06021	0602	351369	138164	287287	212232	211223	272296	330376	199211	311317341	218252	3n	ETT	*24t*
HZ06031	0603	346351363	138180	287287	212232	211231	272280326	330366378	199215	307335343	218252268	3n	ETN	*25t*
HZ06032	0603	346351363	138186	312327	212232269	211231	272280326	330366370	199215	303315347	218250268	3n	ETT	*26t*
HZ06042	0604	351373	138164	287287	212232	211223	272296	330378	199211	311341	218252	2n	ET	*3d*
HZ06041	0604	346355382	138180	287302	212232	211223	272284326	330366374	199215	307315353	218252268	3n	ETN	*23t*
HZ06043	0604	344346351	138180	287307	212232273	211231	276284326	330366378	199215	303335345	218252268	3n	ETN	*27t*
HZ060610	0606	351369	138164	287287	212232	211223	272296	330376	199211	311341	218252	2n	ET	*2d*
HZ060611	0606	351369	138164	287287	212232	211223	272296	330376	199211	311341	218252	2n	ET	*2d*
HZ060613b	0606	351369	138164	287287	212232	211223	272296	330376	199211	311341	218252	2n	ET	*2d*
HZ060616	0606	351369	138164	287287	212232	211223	272296	330376	199211	311341	218252	2n	ET	*2d*
HZ060618	0606	351369	138164	287287	212232	211223	272296	330376	199211	311341	218252	2n	ET	*2d*
HZ060619	0606	351369	138164	287287	212232	211223	272296	330376	199211	311341	218252	2n	ET	*2d*
HZ06062	0606	351369	138164	287287	212232	211223	272296	330376	199211	311341	218252	2n	ET	*2d*
HZ060624	0606	351369	138164	287287	212232	211223	272296	330376	199211	311341	218252	2n	ET	*2d*
HZ060625	0606	351369	138164	287287	212232	211223	272296	330376	199211	311341	218252	2n	ET	*2d*
HZ060628	0606	351369	138164	287287	212232	211223	272296	330376	199211	311341	218252	2n	ET	*2d*
HZ06063	0606	351369	138164	287287	212232	211223	272296	330376	199211	311341	218252	2n	ET	*2d*
HZ06069	0606	351369	138164	287287	212232	211223	272296	330376	199211	311341	218252	2n	ET	*2d*
HZ060622	0606	346351363	138180	287287	212232	211231	272280326	330366378	199215	307335343	218252268	3n	ETN	*25t*
HZ060613a	0606	351369	138164	287287	212232	211223	272296	330376	199211	311317341	218252	3n	ETT	*24t*
HZ060612	0606	351363369	138164	287322	212232265	211223231	272296	330378	199211	311341	218250252	3n	ETT	*28t*
HZ060614	0606	340350367	138164	287317	212232269	211223231	272296	330376388	199211	311333341	218250252	3n	ETT	*29t*
HZ060617	0606	351355369	138164	287287	212232265	211223	272283296	330368376	199211	311333341	218252	3n	ETT	*30t*
HZ060620	0606	351369371	138164	287307	212232236	211223	272296	330376	199211	311337341	218252	3n	ETT	*31t*
HZ060621	0606	351359369	138164	287307	212232	211223	272283296	330376382	199211	311335341	218252	3n	ETT	*32t*
HZ060626	0606	351369371	138164	287317	212232	211223231	272296	330376382	199211	311335341	218252	3n	ETT	*33t*
HZ060627	0606	351359369	138164	287287	212232	211223231	272285296	330376	199211	311333341	218252	3n	ETT	*34t*
HZ06066	0606	351363369	138164	287317	212232269	211223231	272289296	330374376	199211	311337341	218252260	3n	ETT	*35t*
HZ06067	0606	351359369	138164	287307	212232	211223	272296	330376378	199211	311333341	218252	3n	ETT	*36t*
HZ06068	0606	351359369	138164	287287	212232	211223	272285296	330376	199211	311333341	218250252	3n	ETT	*37t*
HZ06061	0606	331336346351	138186	307312	212232	211223	268272280326	330366380388	199219	303329341355	218252264268	4n	ETTN	*18te*

HZ060623	0606	351359369371	138164	287317	212232236261	211223231	272276296	330374376	199211	311337341	218252	4n	ETTN*	*19te*
HZ06064	0606	351363369	138164	287307	212232261	211223	272285296	330376	199211	311317323341	218252	4n	ETTN*	*20te*
HZ06072	0607	313313	138176	317317	212232	211239	276296	330376	199211	303357	218250	2n	ET	*4d*
HZ06075	0607	334340	138164	287287	212232	211239	263270	330378	199211	309347	216250	2n	ET	*5d*
HZ06073	0607	313344	138176	307317	212232	211239	272276296	330374376	199211	303345357	218250252	3n	ETT	*38t*
HZ06074	0607	351369	138164	287307	216232	211239	272275279	330376	199211	311335347	218252264	3n	ETT	*39t*
HZ06082	0608	313313	138176	317317	212232	211239	276296	330376	199211	303357	218250	2n	ET	*4d*
HZ060814	0608	330351	138164	287287	216232	211239	272296	330376	199211	311329	218252	2n	ET	*6d*
HZ060816	0608	330351	138164	287287	212232	211223	272274	330376	199211	311341	216252	2n	ET	*7d*
HZ06086	0608	351369	138164	287287	216232	211239	272274	330376	199211	311347	218252	2n	ET	*8d*
HZ06088	0608	351369	138164	287287	216232	211239	272274	330376	199211	311347	218252	2n	ET	*8d*
HZ06087	0608	351365	138164	287287	216232	211239	272274	330376	199211	311347	218252	2n	ET	*9d*
HZ060819	0608	324336	138138	302312	232232	211211	272283	374374	199199	341357	250272	2n	TT	
HZ060822	0608	351363	138138	302307	232232	211211	274277	366378	199199	345353	250266	2n	TT	
HZ060823	0608	348348	138138	307317	232277	211211	270285	378382	199199	333335	250250	2n	TT	
HZ060811	0608	346355382	138180	287302	212232	211223	272284328	330366374	199215	307315353	218252268	3n	ETN	*19t*
HZ060813	0608	346355382	138180	287302	212232	211223	272284328	330366374	199215	307315353	218252268	3n	ETN	*19t*
HZ060815	0608	346355382	138180	287302	212232	211223	272284326	330366374	199215	307315353	218252268	3n	ETN	*23t*
HZ06083	0608	340350380	138180	287302	212232	211223	272284326	330366374	199216	305311347	218254270	3n	ETN	*20t*
HZ06081	0608	344346351	138180	287307	212232273	211231	276284326	330366378	199215	303335345	218252268	3n	ETN	*27t*
HZ060810	0608	346351371	138180	287307	212232273	211231	277280326	330366378	199219	307315347	218250260	3n	ETN	*40t*
HZ060812	0608	329336351	138164	287307	216232	211239	272279296	330376382	199211	311329347	218250252	3n	ETT	*41t*
HZ060820	0608	351369	138164	287287	216232277	211231239	272274	330376	199211	311347353	218250252	3n	ETT	*42t*
HZ060821	0608	330351359	138164	287307	216232	211239	272283296	330374376	199211	311329353	218250252	3n	ETT	*43t*
HZ060824	0608	309339351	138180	287287	212232	211223	280283326	330366380	199219	307343353	218252	3n	ETN	*44t*
HZ06084	0608	351367369	138164	287307	212232	211223	272285296	330376378	199211	311345	218252	3n	ETT	*45t*
HZ06085	0608	336346351	138186	307312	212232	211223	272280328	330366388	199219	303329341	218264268	3n	ETT	*46t*
HZ06089	0608	336350351	138186	307312	212232	211223	272280326	330366388	199219	303329341	218264268	3n	ETT	*47t*
HZ070110	0701	351369	138164	287287	212232	211223	272296	330376	199211	311341	218252	2n	ET	*2d*
HZ070113	0701	351369	138164	287287	212232	211223	272296	330376	199211	311341	218252	2n	ET	*2d*
HZ070116	0701	351369	138164	287287	212232	211223	272296	330376	199211	311341	218252	2n	ET	*2d*
HZ070119	0701	351369	138164	287287	212232	211223	272296	330376	199211	311341	218252	2n	ET	*2d*
HZ070127	0701	351369	138164	287287	212232	211223	272296	330376	199211	311341	218252	2n	ET	*2d*
HZ070128	0701	351369	138164	287287	212232	211223	272296	330376	199211	311341	218252	2n	ET	*2d*
HZ070144	0701	351369	138164	287287	212232	211223	272296	330376	199211	311341	218252	2n	ET	*2d*
HZ070147	0701	351369	138164	287287	212232	211223	272296	330376	199211	311341	218252	2n	ET	*2d*

HZ07015	0701	351369	138164	287287	212232	211223	272296	330376	199211	311341	218252	2n	ET	*2d*
HZ07012	0701	351365	138164	287287	212232	211223	272296	330376	199211	311341	218252	2n	ET	*11d*
HZ070145	0701	351365	138164	287287	212232	211223	272296	330376	199211	311341	218252	2n	ET	*11d*
HZ070112	0701	313351	138170	307307	212269	211223	277296	330376	199211	303341	218252	2n	ET	*10d*
HZ070117	0701	313351	138170	307307	212269	211223	277296	330376	199211	303341	218252	2n	ET	*10d*
HZ070121	0701	313351	138170	307307	212269	211223	277296	330376	199211	303341	218252	2n	ET	*10d*
HZ070122	0701	313351	138170	307307	212269	211223	277296	330376	199211	303341	218252	2n	ET	*10d*
HZ070123	0701	313351	138170	307307	212269	211223	277296	330376	199211	303341	218252	2n	ET	*10d*
HZ070124	0701	313351	138170	307307	212269	211223	277296	330376	199211	303341	218252	2n	ET	*10d*
HZ070129	0701	313351	138170	307307	212269	211223	277296	330376	199211	303341	218252	2n	ET	*10d*
HZ07013	0701	313351	138170	307307	212269	211223	277296	330376	199211	303341	218252	2n	ET	*10d*
HZ070130	0701	313351	138170	307307	212269	211223	277296	330376	199211	303341	218252	2n	ET	*10d*
HZ070131	0701	313351	138170	307307	212269	211223	277296	330376	199211	303341	218252	2n	ET	*10d*
HZ07014	0701	313351	138170	307307	212269	211223	277296	330376	199211	303341	218252	2n	ET	*10d*
HZ070141	0701	313351	138170	307307	212269	211223	277296	330376	199211	303341	218252	2n	ET	*10d*
HZ07019	0701	313351	138170	307307	212269	211223	277296	330376	199211	303341	218252	2n	ET	*10d*
HZ070126	0701	351369	138164	307307	212232	211223	285296	330376	199211	309345	218252	2n	ET	*12d*
HZ070149	0701	351369	138164	307307	212232	211223	285296	330376	199211	309345	218252	2n	ET	*12d*
HZ07017	0701	351369	138164	307307	212232	211223	285296	330376	199211	309345	218252	2n	ET	*12d*
HZ070136	0701	332336	138138	307307	232232	211211	274283	378378	199199	341353	250252	2n	TT	
HZ070137	0701	336336	138138	307307	232273	211211	272276	376380	199199	333347	250250	2n	TT	
HZ070120	0701	346351359	138180	287287	212232	211231	272280326	330366378	199215	307335343	218252268	3n	ETN	*4t*
HZ070139	0701	346351363	138180	287287	212232	211231	272280326	330366378	199215	307335343	218252268	3n	ETN	*25t*
HZ070133	0701	346355382	138180	287302	212232	211223	272284326	330366374	199215	307315353	218252268	3n	ETN	*23t*
HZ070150	0701	346355382	138180	287302	212232	211223	272284326	330366374	199215	307315353	218252268	3n	ETN	*23t*
HZ070118	0701	346351382	138180	287302	212232	211223	272284326	330366370	199215	307315353	218252268	3n	ETN	*50t*
HZ070132	0701	346351378	138180	287302	212232	211223	272284326	330366374	199215	307315353	218252268	3n	ETN	*52t*
HZ07016	0701	346355378	138180	287302	212232	211223	272284326	330366374	199215	307315353	218252268	3n	ETN	*59t*
HZ07018	0701	346351371	138180	287307	212232273	211231	277280326	330366378	199219	307315347	218250260	3n	ETN	*40t*
HZ070111	0701	309340351	138180	287287	212232	211223	280283326	330366380	199219	307343353	218252	3n	ETN	*48t*
HZ070114	0701	309340351	138180	287287	212232	211223	280283326	330366380	199219	307343353	218252	3n	ETN	*48t*
HZ070151	0701	309340351	138180	287287	212232	211223	280283326	330366380	199219	307343353	218252	3n	ETN	*48t*
HZ070115	0701	334338343	138180	287302	212232	211231	284289326	366378380	199219	307315341	218264268	3n	ETN	*49t*
HZ070125	0701	346355	138180	287302	212232	211223	272284326	330366374	199215	307315353	218252268	3n	ETN	*51t*
HZ070134	0701	313336351	138170	287307314	212269273	211223	277279296	330374376	199211	303341353	218250252	3n	ETT	*53t*
HZ070142	0701	332351369	138164	307307	212232	211223	272285296	330376378	199211	309345353	218250252	3n	ETT	*54t*

HZ070143	0701	346351363	138186	312327	212232	211231	272280326	330366370	199215	303315347	218250268	3n	ETT	*55t*
HZ070146	0701	351359373	138164	287307	212232	211223	272296	330374376	199211	311325341	218252	3n	ETT	*56t*
HZ070148	0701	340351369	138164	287312	212232	211223	272274296	330376378	199211	311341	218250252	3n	ETT	*57t*
HZ070152	0701	309340351	138186	287307	212269277	211223	284297326	330366374	199215	307315337	218252268	3n	ETT	*58t*
HZ07071	0701	336346351	138180	287317	212232265	223231	277284326	330366376	199215	307339353	218250268	3n	ETN	*60t*
HZ07011	0701	336346351374	138180	287302	212232	211223	272284326	330366374376	199215	307315353	218250252268	4n	ETTN*	*21te*
HZ070135	0701	332346355382	138180	287302307	212232273	211223	272276284326	330366374	199215	307315333353	218252268	4n	ETTN	*22te*
HZ070138	0701	336346355382	138180	287302	212232	211223	271284326	330366374378	199215	307315347353	218250252268	4n	ETTN*	*23te*
HZ07021	0702	351373	138164	287287	212232	211223	272296	330376	199211	311341	218252	2n	ET	*13d*
HZ07031	0703	351369	138164	287287	212232	211223	272296	330376	199211	311341	218252	2n	ET	*2d*
HZ070511	0705	351369	138164	287287	212232	211223	272296	330376	199211	311341	218252	2n	ET	*2d*
HZ070515	0705	357390	138164	307307	212232	211239	276276	330374	199211	303333	216252	2n	ET	*18d*
HZ070519	0705	353355	138188	307307	212232	211239	283296	330376	199211	303333	218250	2n	ET	*19d*
HZ070514	0705	344344	138138	307312	232232	211211	272272	378378	199199	325353	250268	2n	TT	
HZ070522	0705	332371	138138	302307	232232	211231	272272	382382	199199	337341	264266	2n	TT	
HZ070524	0705	336336	138138	307312	232269	211231	272276	374378	199199	345345	250252	2n	TT	
HZ070526	0705	351351	138138	287302	232269	211211	272277	374380	199199	341353	250250	2n	TT	
HZ07054	0705	336359	138138	302312	232232	211211	276285	374378	199199	353353	264264	2n	TT	
HZ07058	0705	340351	138138	307307	232232	211211	272276	374378	199199	341353	250252	2n	TT	
HZ070516	0705	346351363	138180	287287	212232	211231	272280326	330366378	199215	307335343	218252268	3n	ETN	*25t*
HZ07055	0705	346351359	138180	287287	212232	211231	274280326	330366378	199215	307335343	218252268	3n	ETN	*80t*
HZ07051	0705	346355382	138180	287302	212232	211223	272284326	330366374	199215	307315353	218252268	3n	ETN	*23t*
HZ070510	0705	346355382	138180	287302	212232	211223	272284326	330366374	199215	307315353	218252268	3n	ETN	*23t*
HZ070517	0705	346355382	138180	287302	212232	211223	272284326	330366374	199215	307315353	218252268	3n	ETN	*23t*
HZ07052	0705	346355382	138180	287302	212232	211223	272284326	330366374	199215	307315353	218252268	3n	ETN	*23t*
HZ07059	0705	346355382	138180	287302	212232	211223	272284326	330366374	199215	307315353	218252268	3n	ETN	*23t*
HZ070521	0705	346355378	138180	287302	212232	211223	272284326	330366374	199215	307315353	218252268	3n	ETN	*59t*
HZ070513	0705	346355382	138180	287302	212232	211223	272284326	330366374	199215	307315353	218252272	3n	ETN	*74t*
HZ070518	0705	346359378	138180	287302	212232	211223	272284326	330366374	199215	307315353	218252268	3n	ETN	*75t*
HZ070520	0705	340350380	138180	287302	212232	211223	272284326	330366374	199216	305311347	218254269	3n	ETN	*76t*
HZ07056	0705	309340351	138186	287307	212269277	211223	284297326	330366374	199215	307315337	218252268	3n	ETT	*58t*
HZ070512	0705	344350353	138180	287287	212232	211231	272280326	330366378	199215	307333341	218252268	3n	ETN	*73t*
HZ070523	0705	336351369	138164	287302	212232	211223	272296	330376382	199211	311341	218250252	3n	ETT	*77t*
HZ070525	0705	340351369	138164	287297	212232	211223	272277296	330374376	199211	311341353	218252	3n	ETT	*78t*
HZ070527	0705	340351353	138188	302307	212232	211239	272283296	330376	199211	303333337	218250252	3n	ETT	*79t*
HZ07053	0705	346359382	138180	287302307	212232	211223	272284326	330366374380	199199	307315353	218250252268	4n	ETTN	*24te*

HZ070618	0706	351369	138164	287287	212232	211223	272296	330376	199211	311341	218252	2n	ET	*2d*
HZ07065	0706	367367	138138	307307	269269	211211	272272	380380	199199	353355	248250	2n	TT	
HZ07062	0706	346351359	138180	287287	212232	211231	272280326	330366378	199215	307335343	218252268	3n	ETN	*4t*
HZ070611	0706	346355382	138180	287302	212232	211223	272284326	330366374	199215	307315353	218252268	3n	ETN	*23t*
HZ07064	0706	346355382	138180	287302	212232	211223	272284326	330366374	199215	307315353	218252268	3n	ETN	*23t*
HZ07069	0706	346355382	138180	287302	212232	211223	272284326	330366374	199215	307315353	218252268	3n	ETN	*23t*
HZ070613	0706	340350380	138180	287302	212232	211223	272284326	330366374	199216	305311347	218254270	3n	ETN	*20t*
HZ070610	0706	309340351	138180	287287	212232	211223	280283326	330366380	199219	307343353	218252	3n	ETN	*48t*
HZ070612	0706	309340351	138180	287287	212232	211223	280283326	330366380	199219	307343353	218252	3n	ETN	*48t*
HZ070614	0706	309340351	138180	287287	212232	211223	280283326	330366380	199219	307343353	218252	3n	ETN	*48t*
HZ07066	0706	309340351	138180	287287	212232	211223	280283326	330366380	199219	307343353	218252	3n	ETN	*48t*
HZ070615	0706	309340348	138180	287287	212232	211223	280283326	330366380	199219	307343353	218252	3n	ETN	*81t*
HZ070621	0706	309338350	138180	287287	212232	211223	280283326	330366380	199219	307341351	218252	3n	ETN	*84t*
HZ070616	0706	309340351	138186	287307	212269277	211223	284297326	330366374	199215	307315337	218252268	3n	ETT	*58t*
HZ07067	0706	309340351	138186	287307	212269277	211223	284297326	330366374	199215	307315337	218252268	3n	ETT	*58t*
HZ07068	0706	309340351	138186	287307	212269277	211223	284297326	330366374	199215	307315337	218252268	3n	ETT	*58t*
HZ070619	0706	309340351	138186	287307	212269277	211223	284297326	330366380	199215	307315337	218252268	3n	ETT	*82t*
HZ070620	0706	346355371	138180	287287	212232	211231	272280326	330366378	199215	307335343	218252268	3n	ETT	*83t*
HZ070622	0706	353373	138176188	307307	212232	211223239	283296300	330376	199211	303311333	218250	3n	EET	*85t*
HZ070623	0706	309340351	138138	287307	212269277	211223	284297326	330366374	199215	307315337	218252268	3n	ETT	*86t*
HZ07063	0706	336351369	138164	307307	212232	211223	272285296	330374376	199211	309341345	218250252	3n	ETT	*87t*
HZ07061	0706	309338350	138180	287312	212232	211223	272280283326	330366380382	199219	307341351	218252272	4n	ETTN*	*25te*
HZ080104	0801	342342	176176	n.a.	212212	223223	n.a.	330330	211211	303311	218220	2n	EE	
HZ080120	0801	353353	176178	n.a.	212212	223239	n.a.	330330	211211	303303	218218	2n	EE	
HZ080133	0801	342353	176176	n.a.	212212	223239	n.a.	330330	211211	309313	218220	2n	EE	
HZ080140	0801	357373	164164	n.a.	212212	239239	n.a.	330330	211211	303311	218218	2n	EE	
HZ080141	0801	342353	164176	n.a.	212212	223239	n.a.	330330	211211	309311	218220	2n	EE	
HZ080143	0801	342353	176178	n.a.	212212	223223	n.a.	330330	211211	303313	218218	2n	EE	
HZ080145	0801	342342	176188	n.a.	212212	239239	n.a.	330330	211211	303309	218220	2n	EE	
HZ080147	0801	342353	176176	n.a.	212212	223239	n.a.	330330	211211	303303	218218	2n	EE	
HZ080148	0801	353357	176176	n.a.	212212	223223	n.a.	330330	211211	303311	220220	2n	EE	
HZ080155	0801	342365	176188	n.a.	212212	223239	n.a.	330330	211211	303303	218220	2n	EE	
80130	0801	313351353	138170188	302302	212232	211223	n.a.	330376	199211	303311345	218220258	3n	EET	*1t*
80131	0801	313351353	138170188	302302	212232	211223	n.a.	330376	199211	303311345	218220258	3n	EET	*1t*
80137	0801	313351353	138170188	302302	212232	211223	n.a.	330376	199211	303311345	218220258	3n	EET	*1t*
80144	0801	313351353	138170188	302302	212232	211223	n.a.	330376	199211	303311345	218220258	3n	EET	*1t*

80103	0801	309346351	138180186	287287	212232	211223231	n.a.	330366	215219	303307315	218268	3n	EEN	*9t*
80136	0801	309346351	138180186	287287	212232	211223231	n.a.	330366	215219	303307315	218268	3n	EEN	*9t*
80101	0801	313351353	138164188	302302	212232	211223239	n.a.	330376	199211	311335	218220252	3n	EET	*88t*
80108	0801	313351353	138164188	302302	212232	211223239	n.a.	330376	199211	311335	218220252	3n	EET	*88t*
80113	0801	313351353	138164188	302302	212232	211223239	n.a.	330376	199211	311335	218220252	3n	EET	*88t*
80114	0801	313351353	138164188	302302	212232	211223239	n.a.	330376	199211	311335	218220252	3n	EET	*88t*
80125	0801	313351353	138164188	302302	212232	211223239	n.a.	330376	199211	311335	218220252	3n	EET	*88t*
80149	0801	313351353	138164188	302302	212232	211223239	n.a.	330376	199211	311335	218220252	3n	EET	*88t*
80151	0801	313351353	138164188	302302	212232	211223239	n.a.	330376	199211	311335	218220252	3n	EET	*88t*
80102	0801	313338351	138176188	302302	212232	211223	n.a.	330376	199211	303311335	218252	3n	EET	*89t*
80135	0801	313338351	138176188	302302	212232	211223	n.a.	330376	199211	303311335	218252	3n	EET	*89t*
80105	0801	313351353	138164188	307307	212232	211223239	n.a.	330376	199211	311335	218220252	3n	EET	*90t*
80106	0801	313351	138178188	302302	212232	211223239	n.a.	330376	199211	303311345	218252	3n	EET	*91t*
80112	0801	313351353	138178188	302302	212232	211223239	n.a.	330376	199211	303311345	218252	3n	EET	*94t*
80126	0801	313351353	138178188	302302	212232	211223239	n.a.	330376	199211	303311345	218252	3n	EET	*94t*
80139	0801	313351353	138178188	302302	212232	211223239	n.a.	330376	199211	303311345	218252	3n	EET	*94t*
80156	0801	313351353	138178188	302302	212232	211223239	n.a.	330376	199211	303311345	218252	3n	EET	*94t*
80158	0801	313351353	138178188	302302	212232	211223239	n.a.	330376	199211	303311345	218252	3n	EET	*94t*
80107	0801	313351353	138188	302302	212232	211223	n.a.	330376	199211	303311345	218220258	3n	EET	*92t*
80115	0801	313346382	138170186	287287	212232	223223	n.a.	330366	211219	309315	216218274	3n	EET	*95t*
80110	0801	313349382	138170186	287287	212232	223223	n.a.	330366	211219	309315	216218274	3n	EET	*93t*
80111	0801	313349382	138170186	287287	212232	223223	n.a.	330366	211219	309315	216218274	3n	EET	*93t*
80121	0801	313349382	138170186	287287	212232	223223	n.a.	330366	211219	309315	216218274	3n	EET	*93t*
80117	0801	313351353	138178188	292292	212232	211223239	n.a.	330376	199211	303311335	218220252	3n	EET	*96t*
80124	0801	313351353	138178188	292292	212232	211223239	n.a.	330376	199211	303311335	218220252	3n	EET	*96t*
80132	0801	313351353	138178188	292292	212232	211223239	n.a.	330376	199211	303311335	218220252	3n	EET	*96t*
80128	0801	309346359	138180186	287287	212232	211223231	n.a.	330366	215219	303307315	218268	3n	EET	*97t*
HZ080109	0801	313342351353	138170176188	302	212232	211223239	n.a.	330376	199211	303311313345	218220258	4n	EEET	*26te*
HZ080142	0801	309346351353	138178186	287	212232	211223231239	n.a.	330366	211215219	303307315	218268	4n	EEEN	*27te*
HZ080146	0801	313351353	138164188	302	212232	211223239	n.a.	330376	199211	303311335	216218220252	4n	EEET	*28te*
HZ080150	0801	309342346351	138178186	287	212232	211223231	n.a.	330366	211215219	303307313315	218268	4n	EEEN*	*29te*
HZ080152	0801	309346351353	138176180186	287	212232	211223239	n.a.	330366	211215219	303307315	218268	4n	EEEN*	*30te*
HZ080153	0801	313342351353	138164176188	302	212232	211223239	n.a.	330376	199211	311335	218220252	4n	EEET	*31te*
HZ080154	0801	309346351353	138180186	287	212232	211223231239	n.a.	330366	211215219	303307315	218268	4n	EEEN	*32te*
HZ080157	0801	313342351353	138164178188	302	212232	211223239	n.a.	330376	199211	303311345	218252	4n	EEET	*33te*
HZ080159	0801	313342351353	138164178188	292	212232	211223239	n.a.	330376	199211	303311335	216218220252	4n	EEEN	*34te*

HZ080160	0801	313342351353	138178188	292	212232	211223239	n.a.	330376	199211	303311313335	218220252	4n	EEET	*35te*
80404	0804	309346351	138180186	287287	212232	211223231	n.a.	330366	215219	303307315	218268	3n	EEN	*9t*
80405	0804	309346351	138180186	287287	212232	211223231	n.a.	330366	215219	303307315	218268	3n	EEN	*9t*
80406	0804	309346351	138180186	287287	212232	211223231	n.a.	330366	215219	303307315	218268	3n	EEN	*9t*
80407	0804	309346351	138180186	287287	212232	211223231	n.a.	330366	215219	303307315	218268	3n	EEN	*9t*
80410	0804	309346355	138180186	287287	212232	211223231	n.a.	330366	215219	303307315	218268	3n	EEN	*99t*
80411	0804	309346355	138180186	287287	212232	211223231	n.a.	330366	215219	303307315	218268	3n	EEN	*99t*
80408	0804	313351353	138170188	302302	212232	211223	n.a.	330376	199211	303311345	218220	3n	EET	*98t*
80412	0804	336349351	138186	307312	212232	211223	n.a.	330366388	199219	303333341	218264268	3n	EET	*100t*
HZ080401	0804	309342346351	138176180186	287	212232	211223239	n.a.	330366	211215219	303307315	218268	4n	EEEN*	*36te*
HZ080402	0804	344346351359	138180	287312	212232269	211231	n.a.	330366378	199215	307335343345	218250252268	4n	ETTN*	*37te*
HZ080403	0804	346351359	138180	287307	212232	211231	n.a.	330366378	199215	307333335343	218250252268	4n	ETTN*	*38te*
HZ080409	0804	309350355	138164180186	287	212232	211223231239	n.a.	330366	215219	303307315	218268	4n	EEEN	*39te*
HZ090101	0901	348378	138138	287302	232232	211211	n.a.	374374	199199	353353	250252	2n	TT	
HZ090102	0901	348348	138138	287307	232232	211211	n.a.	374378	199199	353353	250260	2n	TT	
HZ090301	0903	340344	138138	307312	232269	211231	n.a.	378380	199199	353353	248250	2n	TT	
HZ090302	0903	355367	138138	307307	232269	211211	n.a.	382382	199199	333353	252252	2n	TT	
HZ090303	0903	340348	138138	307312	232269	231231	n.a.	378378	199199	337353	250252	2n	TT	
HZ090304	0903	344348	138138	307312	232232	211211	n.a.	378380	199199	341351	250252	2n	TT	
HZ090305	0903	344344	138138	312312	232269	211211	n.a.	380380	199199	357357	250256	2n	TT	
HZ090306	0903	344348	138138	307307	269269	211211	n.a.	378378	199199	333357	252256	2n	TT	
HZ090307	0903	340344	138138	307307	256261	211211	n.a.	374378	199199	341353	252256	2n	TT	
HZ090308	0903	348367	138138	312312	232232	211211	n.a.	370370	199199	351351	250264	2n	TT	
HZ090309	0903	344344	138138	307317	232232	211231	n.a.	370378	199199	333353	264264	2n	TT	
HZ090310	0903	336336	138138	302312	232232	211231	n.a.	378380	199199	347357	252256	2n	TT	
HZ090311	0903	348348	138138	307307	232273	211231	n.a.	378380	199199	353355	250252	2n	TT	
HZ090312	0903	348367	138138	307312	232273	211211	n.a.	370380	199199	335355	250250	2n	TT	
HZ090313	0903	340340	138138	307312	232256	211231	n.a.	370380	199199	333333	250250	2n	TT	
HZ090314	0903	340355	138138	307307	232269	211211	n.a.	370374	199199	333341	250252	2n	TT	
