# Supplementary material for: Dynamic Formation of Asexual Diploid and Polyploid Lineages: Multilocus Analysis of Cobitis Reveals the Mechanisms Maintaining the Diversity of Clones
Source: PLoS One. 2012 Sep 20;7(9):e45384. doi: 10.1371/journal.pone.0045384 (PMC3447977; doi:10.1371/journal.pone.0045384)
Supplement: Table S4 — Allelic diversity within populations of microsatellite loci for diploid specimens. (PDF) [file pone.0045384.s005.pdf]

Table S4. Allelic diversity within populations of microsatellite loci for diploid specimens.

Locality ID	Locus	Sample size	No. of Different Alleles	Observed Heterozygosity
0502	cota_006	2	4	1
0502	cota_010	2	2	0.5
0502	cota_027	2	1	0
0502	cota_032	2	4	1
0502	cota_033	2	2	0.5
0502	cota_037	2	3	1
0502	cota_041	2	4	1
0502	cota_068	2	2	0.5
0502	cota_093	2	4	1
0502	cota_111	2	2	0.5
0503	cota_006	1	1	0
0503	cota_010	1	1	0
0503	cota_027	1	1	0
0503	cota_032	1	1	0
0503	cota_033	1	2	1
0503	cota_037	1	1	0
0503	cota_041	1	2	1
0503	cota_068	1	1	0
0503	cota_093	1	2	1
0503	cota_111	1	2	1
0507	cota_006	9	5	0.556
0507	cota_010	9	5	0.778
0507	cota_027	0	0	0
0507	cota_032	9	1	0
0507	cota_033	9	2	0.556
0507	cota_037	9	6	0.778
0507	cota_041	9	1	0
0507	cota_068	9	1	0
0507	cota_093	9	3	0.556
0507	cota_111	9	3	0.444
0508	cota_006	6	5	0.5
0508	cota_010	6	6	0.833
0508	cota_027	0	0	0
0508	cota_032	6	1	0
0508	cota_033	6	2	0.667
0508	cota_037	6	6	0.833
0508	cota_041	6	2	0.167
0508	cota_068	6	1	0
0508	cota_093	6	4	0.833
0508	cota_111	6	3	0.5
0509	cota_006	7	6	0.571
0509	cota_010	7	4	0.857
0509	cota_027	0	0	0
0509	cota_032	7	1	0
0509	cota_033	7	2	0.286
0509	cota_037	7	8	1
0509	cota_041	7	2	0.143
0509	cota_068	7	1	0
0509	cota_093	7	4	1

0509	cota_111	7	3	0.571
0511	cota_006	6	5	0.833
0511	cota_010	6	7	1
0511	cota_027	0	0	0
0511	cota_032	6	1	0
0511	cota_033	6	2	0.333
0511	cota_037	6	4	0.833
0511	cota_041	6	2	0.167
0511	cota_068	6	1	0
0511	cota_093	6	2	0.333
0511	cota_111	6	2	0.167
0513	cota_006	33	19	0.848
0513	cota_010	33	1	0
0513	cota_027	33	6	0.545
0513	cota_032	33	6	0.515
0513	cota_033	33	2	0.576
0513	cota_037	33	9	0.909
0513	cota_041	33	11	0.758
0513	cota_068	33	1	0
0513	cota_093	33	14	0.909
0513	cota_111	33	10	0.879
0515	cota_006	3	5	1
0515	cota_010	3	2	0.333
0515	cota_027	3	4	0.667
0515	cota_032	3	3	0.667
0515	cota_033	3	2	0.333
0515	cota_037	3	4	0.667
0515	cota_041	3	5	1
0515	cota_068	3	2	0.333
0515	cota_093	3	6	1
0515	cota_111	3	4	1
0604	cota_006	1	2	1
0604	cota_010	1	2	1
0604	cota_027	1	1	0
0604	cota_032	1	2	1
0604	cota_033	1	2	1
0604	cota_037	1	2	1
0604	cota_041	1	2	1
0604	cota_068	1	2	1
0604	cota_093	1	2	1
0604	cota_111	1	2	1
0606	cota_006	12	2	1
0607	cota_010	12	2	1
0608	cota_027	12	1	0
0609	cota_032	12	2	1
0610	cota_033	12	2	1
0611	cota_037	12	2	1
0612	cota_041	12	2	1
0613	cota_068	12	2	1
0614	cota_093	12	2	1
0615	cota_111	12	2	1
0607	cota_006	2	3	0.5
0607	cota_010	2	3	1
0607	cota_027	2	2	0
0607	cota_032	2	2	1
0607	cota_033	2	2	1

0607	cota_037	2	4	1
0607	cota_041	2	3	1
0607	cota_068	2	2	1
0607	cota_093	2	4	1
0607	cota_111	2	3	1
0608	cota_006	9	9	0.778
0608	cota_010	9	3	0.667
0608	cota_027	9	5	0.333
0608	cota_032	9	4	0.778
0608	cota_033	9	3	0.667
0608	cota_037	9	8	1
0608	cota_041	9	6	0.889
0608	cota_068	9	2	0.667
0608	cota_093	9	10	1
0608	cota_111	9	6	0.889
0701	cota_006	29	6	0.966
0701	cota_010	29	3	0.931
0701	cota_027	29	2	0
0701	cota_032	29	4	0.966
0701	cota_033	29	2	0.931
0701	cota_037	29	7	1
0701	cota_041	29	4	0.966
0701	cota_068	29	2	0.931
0701	cota_093	29	8	1
0701	cota_111	29	3	0.966
0702	cota_006	1	2	1
0702	cota_010	1	2	1
0702	cota_027	1	1	0
0702	cota_032	1	2	1
0702	cota_033	1	2	1
0702	cota_037	1	2	1
0702	cota_041	1	2	1
0702	cota_068	1	2	1
0702	cota_093	1	2	1
0702	cota_111	1	2	1
0703	cota_006	1	2	1
0703	cota_010	1	2	1
0703	cota_027	1	1	0
0703	cota_032	1	2	1
0703	cota_033	1	2	1
0703	cota_037	1	2	1
0703	cota_041	1	2	1
0703	cota_068	1	2	1
0703	cota_093	1	2	1
0703	cota_111	1	2	1
0601	cota_006	26	7	0.962
0601	cota_010	26	2	0.962
0601	cota_027	26	2	0
0601	cota_032	26	3	1
0601	cota_033	26	3	1
0601	cota_037	25	3	1
0601	cota_041	26	4	1
0601	cota_068	26	2	0.962
0601	cota_093	26	4	1
0601	cota_111	26	4	1
0705	cota_006	9	12	0.667

0705	cota_010	9	3	0.333
0705	cota_027	9	4	0.556
0705	cota_032	9	3	0.556
0705	cota_033	9	4	0.556
0705	cota_037	9	6	0.667
0705	cota_041	9	6	0.778
0705	cota_068	9	2	0.333
0705	cota_093	9	8	0.778
0705	cota_111	9	7	0.778
0706	cota_006	2	3	0.5
0706	cota_010	2	2	0.5
0706	cota_027	2	2	0
0706	cota_032	2	3	0.5
0706	cota_033	2	2	0.5
0706	cota_037	2	2	0.5
0706	cota_041	2	3	0.5
0706	cota_068	2	2	0.5
0706	cota_093	2	4	1
0706	cota_111	2	4	1
0801	cota_006	10	5	0.7
0801	cota_010	10	4	0.5
0801	cota_027	0	0	0
0801	cota_032	10	1	0
0801	cota_033	10	2	0.5
0801	cota_037	0	0	0
0801	cota_041	10	1	0
0801	cota_068	10	1	0
0801	cota_093	10	4	0.7
0801	cota_111	10	2	0.5
0901	cota_006	2	2	0.5
0901	cota_010	2	1	0
0901	cota_027	2	3	1
0901	cota_032	2	1	0
0901	cota_033	2	1	0
0901	cota_037	0	0	0
0901	cota_041	2	2	0.5
0901	cota_068	2	1	0
0901	cota_093	2	1	0
0901	cota_111	2	3	1
0903	cota_006	14	6	0.643
0903	cota_010	14	1	0
0903	cota_027	14	4	0.5
0903	cota_032	14	5	0.643
0903	cota_033	14	2	0.357
0903	cota_037	0	0	0
0903	cota_041	14	5	0.643
0903	cota_068	14	1	0
0903	cota_093	14	9	0.714
0903	cota_111	14	5	0.714
