# Supplementary material for: Dynamic Formation of Asexual Diploid and Polyploid Lineages: Multilocus Analysis of Cobitis Reveals the Mechanisms Maintaining the Diversity of Clones
Source: PLoS One. 2012 Sep 20;7(9):e45384. doi: 10.1371/journal.pone.0045384 (PMC3447977; doi:10.1371/journal.pone.0045384)
Supplement: Table S5 — Microsatellite allelic diversity for diploid biotypes. (PDF) [file pone.0045384.s006.pdf]

Table S5. Microsatellite allelic diversity for diploid biotypes.

Species/ hybrid	Locus	Sample size	No. of Different Alleles	Observed Heterozygosity
EE	cota_006	38	10	0.632
EE	cota_010	38	8	0.763
EE	cota_027	NA	NA	NA
EE	cota_032	38	1	0
EE	cota_033	38	2	0.474
EE	cota_037	28	10	0.857
EE	cota_041	38	2	0.079
EE	cota_068	38	1	0
EE	cota_093	38	5	0.684
EE	cota_111	38	3	0.447
ET	cota_006	81	14	0.975
ET	cota_010	81	5	1
ET	cota_027	81	3	0
ET	cota_032	81	4	1
ET	cota_033	81	4	1
ET	cota_037	81	10	0.988
ET	cota_041	81	4	1
ET	cota_068	81	2	1
ET	cota_093	81	9	1
ET	cota_111	81	5	1
TT	cota_006	66	24	0.712
TT	cota_010	66	1	0
TT	cota_027	66	7	0.561
TT	cota_032	66	7	0.5
TT	cota_033	66	2	0.424
TT	cota_037	49	10	0.837
TT	cota_041	66	12	0.712
TT	cota_068	66	1	0
TT	cota_093	66	16	0.833
TT	cota_111	66	13	0.803
