# Supplementary material for: Dynamic Formation of Asexual Diploid and Polyploid Lineages: Multilocus Analysis of Cobitis Reveals the Mechanisms Maintaining the Diversity of Clones
Source: PLoS One. 2012 Sep 20;7(9):e45384. doi: 10.1371/journal.pone.0045384 (PMC3447977; doi:10.1371/journal.pone.0045384)
Supplement: Text S1 — Detailed description of methods applied to delimit the clonal lineages and to estimate the distribution of clonal ages. (DOC) [file pone.0045384.s007.doc]

**Text S1.**

**Delimitation of clonal lineages**

We used simulation to obtain the expected distribution of pair-wise distances among MLGs under the assumption that each MLG represents an independent clone. In the case of diploids, we simulated a population of 500 diploid hybrids by selecting random parents from the *C. taenia* and *C. elongatoides* datasets. In the case of triploids, we randomly selected the maternal ancestor as either a simulated or an observed diploid hybrid, while the paternal ancestor was selected from either *C. taenia* or *C. elongatoides* datasets.

Simulated distributions of pairwise distances were fitted by empirical distribution functions using R software [1]. This software allowed us to assign any value of observed pairwise distances between two MLG by using a probability value, and decide whether such pairwise distances are significantly small to infer that both MLG belong to the same clone. To correct for multiple comparisons, we applied the sequential Bonferroni’s correction in a following way: For each MLG we selected its closest neighbor, and tested whether the probability of their distance was less than the chosen alpha-level divided by the number of samples. If so, we checked whether the probability of distance between the second closest MLG was less than alpha/(Number of samples–1) and so on, until the series produced no significant results.

All MLGs that had a significantly small distance were considered representatives of the same MLL. Finally, to confirm our assignment in diploids, we further calculated the P_sex_ on the set of loci that were identical among putative members of the same MLL [2].

**Relative estimation of clonal ages**

To evaluate the suitability of the *dist.mut* and *dist.bp* indices used to estimate the age of clones, we used the simulations by using an Individual-Based Model (IBM).

We started the model with two reproductively isolated species, each divided into two regions (demes) interconnected by individual migration rate *m*. Each population was composed of *n* sexually reproducing diploids, each with 10 unlinked microsatellite loci. Initially, each locus started in the homozygous state. We modeled a process of mitosis and recombination during the 1^st^ meiotic division. Alleles of size 1 repeat were not allowed to mutate, and hence this allelic class was considered to be in an absorbing state *sensu* [3]. New alleles occurred by mutations; either following the stepwise mutation model (SMM) or mutating to any possible state (K-mutation model; KAM). The possibility of homoplasy is therefore inherent in our model. We ran 3 sets of simulation, 1 with SSM only, the others with a KAM frequency of 5% and 10% respectively [4]. Combining the 2 haploid sets with randomly chosen individuals from the previous generation created new sexual individuals within both populations. We allowed the sexual populations to evolve for 8N generations to reach equilibrium.

After the initial period, random pairs of individuals were selected from each sexual species as maternal and paternal ancestor of the new hybrid clonal population. The parental sexual pairs were chosen so that we either combined individuals from the first region in species 1 and the first region in the species 2 or we combined individuals from the second regions of both species. Immediately after the hybridization event, the clonal population had reached its stable population size of *n* individuals, and both sexual species were allowed to evolve in parallel for 40,000 generations. In case of triploids, we combined haploid paternal set of alleles with the entire maternal genome. For every 200^th^ generations we calculated the *dist.mut* and *dist.bp,* indices. The distances from sexual progenitor were measured so that we compared clones against the sexual individuals from the regions, where the clones originated (i.e. simulating the case when we sample the true parental populations) as well as from allopatric demes of both species (simulating the case when the clones are of unknown, probably allopatric, geographical origin). We ran the simulation with varying migration rates (*m*), so that the product *n*m* covers the values from 1 to 0.01migrants per generation. Mutation rates (μ) varied so that we covered the entire biologically plausible range, spanning from 10^-6^ to 10^-2^. For each value of *m* and θ (=4 *n**μ), we ran the simulation 500 times.

LITERATURE CITED

1. R Development Core Team: R: 2008. A language and environment for statistical computing. R Foundation for Statistical Computing: Vienna, Austria. [http://www.R-project.org](http://www.R-project.org/).
2. Arnaud-Haond, S., C. M. Duarte, F. Alberto, and E.A. Serrao. 2007. Standardizing methods to address clonality in population studies. Mol. Ecol. 16:5115–39.
3. Samadi, S., F., Erard, A. Estoup, and P. Jarne. 1998. The influence of mutation, selection and reproductive systems on microsatellite variability: a simulation approach. Genet. Res. 7:213–22.
4. Balloux, F., H. Brünner, N. Lugon-Moulin, J. Hausser, and J. Goudet. 2000. Microsatellites can be misleading: an empirical and simulation study. Evolution 54:1414–22.
